# Transcriptome-wide functional characterization reveals novel relationships among differentially expressed transcripts in developing soybean embryos

**DOI:** 10.1186/s12864-015-2108-x

**Published:** 2015-11-14

**Authors:** Delasa Aghamirzaie, Dhruv Batra, Lenwood S. Heath, Andrew Schneider, Ruth Grene, Eva Collakova

**Affiliations:** Genetics, Bioinformatics and Computational Biology Program, Virginia Tech, Blacksburg, VA 24061 USA; Bradley Department of Electrical and Computer Engineering, Virginia Tech, Blacksburg, VA 24061 USA; Department of Computer Science, Virginia Tech, Blacksburg, VA 24061 USA; Department of Plant Pathology, Physiology, and Weed Science, Virginia Tech, Blacksburg, VA 24061 USA

## Abstract

**Background:**

Transcriptomics reveals the existence of transcripts of different coding potential and strand orientation. Alternative splicing (AS) can yield proteins with altered number and types of functional domains, suggesting the global occurrence of transcriptional and post-transcriptional events. Many biological processes, including seed maturation and desiccation, are regulated post-transcriptionally (e.g., by AS), leading to the production of more than one coding or noncoding sense transcript from a single locus.

**Results:**

We present an integrated computational framework to predict isoform-specific functions of plant transcripts. This framework includes a novel plant-specific weighted support vector machine classifier called CodeWise, which predicts the coding potential of transcripts with over 96 % accuracy, and several other tools enabling global sequence similarity, functional domain, and co-expression network analyses. First, this framework was applied to all detected transcripts (103,106), out of which 13 % was predicted by CodeWise to be noncoding RNAs in developing soybean embryos. Second, to investigate the role of AS during soybean embryo development, a population of 2,938 alternatively spliced and differentially expressed splice variants was analyzed and mined with respect to timing of expression. Conserved domain analyses revealed that AS resulted in global changes in the number, types, and extent of truncation of functional domains in protein variants. Isoform-specific co-expression network analysis using ArrayMining and clustering analyses revealed specific sub-networks and potential interactions among the components of selected signaling pathways related to seed maturation and the acquisition of desiccation tolerance. These signaling pathways involved abscisic acid- and FUSCA3-related transcripts, several of which were classified as noncoding and/or antisense transcripts and were co-expressed with corresponding coding transcripts. Noncoding and antisense transcripts likely play important regulatory roles in seed maturation- and desiccation-related signaling in soybean.

**Conclusions:**

This work demonstrates how our integrated framework can be implemented to make experimentally testable predictions regarding the coding potential, co-expression, co-regulation, and function of transcripts and proteins related to a biological process of interest.

**Electronic supplementary material:**

The online version of this article (doi:10.1186/s12864-015-2108-x) contains supplementary material, which is available to authorized users.

## Background

Seed maturation and induction of dormancy represent essential stages in soybean seed development that are triggered through highly coordinated signaling and metabolic pathways within the seed maturation and desiccation programs. LEAFY COTYLEDON (LEC) 1 and transcription factors (TFs) containing the B3 DNA-binding domain, namely LEC2, ABSCISIC ACID INSENSITIVE (ABI) 3, and FUSCA (FUS) 3, are key regulators of seed filling, commonly called “the B3 regulatory network” [1, 2]. Their mutual interactions and interactions with their targets and components of phytohormone-mediated signaling connect these TFs within the well-studied B3 regulatory network to developmental and metabolic processes leading to the synthesis and accumulation of seed storage compounds. During late seed filling, maturing seeds acquire desiccation tolerance (DT) and dormancy, as the water content decreases, primarily through abscisic acid (ABA)-mediated signaling [1, 3–5]. Seed filling-, desiccation-, and dormancy-related processes are regulated at both the transcriptional and post-transcriptional levels. To gain a basic understanding of these regulatory processes, it is important to identify additional regulatory molecules, e.g., proteins and RNA, involved in the seed maturation developmental program, which can be achieved through transcriptomics in conjunction with bioinformatics analyses.

High-throughput RNA sequencing (RNA-Seq) reveals high transcriptional activity in unannotated and annotated regions of genomes in various organisms, resulting in the discovery of many previously unknown transcripts [6, 7]. Alternative splicing (AS) is a major source of this transcript diversity, as a single gene can encode multiple transcripts. These transcripts can be coding or noncoding, genic or intergenic, and sense or antisense. Coding transcripts are translated into proteins or regulatory peptides that can contain, or lack known domains important for function, regulation, interaction with other molecules, and subcellular localization [8, 9]. In contrast, noncoding transcripts, including long noncoding RNAs (lncRNA) and long intergenic noncoding RNAs (lincRNA) can act directly as regulators [6, 7, 10, 11]. These noncoding RNAs (ncRNAs) perform their regulatory functions through transcriptional interference, sense and antisense hybridization, interactions with RNA-binding proteins, and/or serving as precursors for small regulatory RNAs [7, 11]. Noncoding transcripts have been reported to be involved in the regulation of development and in responses to stress in plants [12–14]. To date, specific plant lncRNAs have been implicated in the regulation of flowering, response to cold, root meristem development, and modulation of AS [15].

High-throughput experimental testing to predict the functions of newly identified transcripts is not possible. As a first step towards future experimentation, the functions of coding and noncoding transcripts can be inferred computationally by integrating several approaches, such as functional annotation based on sequence similarity, global functional domain analyses, determining the coding potential of transcripts, co-expression analyses, and the construction of hypothetical regulatory networks. Because transcripts can be coding or noncoding, determining their coding potential is a necessary step towards their functional characterization. Identification of conserved domains within newly identified coding sequences using well-established tools such as InterPro [16] and Batch Conserved Domain (CD) Search [17] is important for *in silico* function prediction.

Several tools have been developed for predicting the coding potential of individual transcripts primarily in animal systems, including Coding Potential Calculator (CPC) [18], Coding Potential Assessment Tool (CPAT) [19], PhyloCSF [20], and iSeeRNA [21]. These tools rely upon sequence similarity and open reading frame (ORF) length to distinguish between coding and noncoding transcripts [10, 22]. However sequence similarity and ORF length alone lack sufficient power to accurately distinguish between coding and noncoding RNAs (ncRNA). Additional features, such as the presence of conserved functional domains, GC content, and the free energy of RNA secondary structure, are needed to improve the detection accuracy of ncRNAs [12, 23, 24]. To our knowledge, there are currently no comparable tools available to globally characterize coding and ncRNAs specifically in plants.

Here we present the development and implementation of a transcriptome-wide computational framework that combines high-throughput information with bioinformatics tools to predict potential functions and novel associations among transcripts and inferred proteins. Co-expression-related guilt-by-associations, timing of expression, sequence similarity, presence of functional domains in protein variants, and coding potential of transcripts were each used to infer possible function. The framework includes (i) a pipeline for global analysis of functional domains in proteins, and (ii) CodeWise, an accurate support vector machine (SVM) classifier that uses several features to predict the coding potential of transcripts. This framework was applied to an existing data set related to seed filling and early desiccation stages in developing soybean embryos [25, 26]. We mined this data set extensively in the context of AS events and (i) the coding potential of transcripts, (ii) the presence or absence of functional domains, (iii) similarity to Arabidopsis proteins, and (iv) timing and patterns of expression, including co-expression network analysis, during soybean embryo development. Highly connected nodes within the co-expression network (hubs) connecting the majority of transcripts expressed during the desiccation phase were identified. Hypothetical ABA- and FUS3-related signaling pathways focusing specifically on signaling components subjected to AS and related to soybean seed filling and acquisition of DT are also presented and discussed.

## Methods

### Definition of terms

Common terms used in this study are defined in Table 1.

**Table 1:** Glossary of common terms that were used in this study

Term	Definition
Degree of connectivity	Identification of how well a node is connected in a network. For example, if a network has 10 nodes and a node is connected to 5 nodes, it’s degree of connectivty is 0.5.
Desiccation tolerance (DT) phase	The last phase in developing embryos characterized by loss of water, a sharp increase of desiccation-related metabolites and transcripts, and acquisition of DT in yellow embryos at day 55.
Differentially expressed transcript	A transcript that was significantly differentially expressed at at least one time point compared with previous time point (FDR < 0.05).
Domain categorization	The domain composition of SV-pairs were compared and categorized into similar domains, no known domain, and disparate domains.
Early maturation phase	This first phase in seed filling is characterized by an initial decrease in metabolites and cell-division-related transcript levels and the onset of accumulation of seed storage compounds.
Expressed transcripts	A transcript was defined as expressed if the sum of its FPKM values across the time course was greater than one.
Hub	Highly connected nodes in a network (nodes with the highest degree of connectivity).
Mid-to-late maturation phase	This phase in embryo development is characterized by a stable accumulation of seed storage compounds.
Nearest neighbors	Nodes directly connected through individual edges to a single node of interest.
Regulon	A group of transcripts known to be targets of a common TF. For example, a group of transcripts known to be targets of ABI3, is called the ABI3 regulon.
Soybean developmental stages	Three major developmental stages (early maturation, mid-to-late maturation, and DT) defined in this report on the basis of changes in the levels of relevant metabolites, seed storage compounds, and transcripts in developing soybean embryos.
Splice variant (SV)	Transcripts that are products of the same precursor mRNA.
Sub-network	A group of nodes and edges that are part of a larger network. For example, a node with all its nearest neighbors comprising the members of the FUS3 regulon is a sub-network.
Super-cluster	Clusters of transcripts with similar expression profiles grouped according to predefined soybean developmental stages.
SV group	A group containing at least two SVs of a gene both of which were significantly differentially expressed during embryo development. For example, gene X has three SVs (X.1, X.2, X.3), and X.1 and X.3 showed changes in transcript levels and X.2 was stably expressed in developing embryos. X.1 and X.3 belong to the same SV group representing gene X.

### Analysis of RNA-Seq data and identification of differentially expressed transcripts

Our RNA-Seq data set (GEO accession number GSE46153) includes ten time points with three biological replicates per time point, representing the phases of soybean embryo development from the onset of seed filling to the onset of seed desiccation. Read mapping, transcriptome assembly, and differential expression analyses were done using Tophat2, Cufflinks, and Cuffdiff2 available in the Tuxedo Suite [27] RNA-Seq pipeline [25, 26]. The *Glycine max* reference genome (version 189) was used to guide transcriptome assembly, which yielded 39,191 known and 64,005 novel expressed transcripts. A transcript was defined as expressed if the sum of its FPKM (fragments per kilobase of exon per million fragments mapped) values across the time course was greater than 1. Based on the Cuffdiff2 results and temporal differential expression analysis at the isoform level, 17,181 transcripts were significantly differentially expressed during at least one time point when compared to the previous time point (false discovery rate (FDR) < 0.05). Based on further categorization, 2,938 out of 17,181 transcripts were also alternatively spliced and originated from 1,393 genes, meaning that for each of these genes, at least two differentially expressed splice variants (SVs) were identified. Nucleotide sequences of newly assembled transcripts were extracted and assembled using an in-house Python program to parse the transcriptome reference output by Cuffmerge (merged.gtf) from the soybean genome. Class codes used are a set of 12 Cuffcompare transcript codes proposed by [28]. Novel transcripts appeared as novel SVs of known genes (transcript classes “j”, “o”, and “c”), as well as in intergenic (transcript classes “-” and “u”) and antisense (transcript classes “x” and “s”) classes. The term “transcript” is used as a general term and includes all types of detected transcripts as opposed to SVs that are defined as transcripts produced from the same premature messenger RNA (pre-mRNA). The nomenclature for novel SVs was adapted from [25]. For example, if a gene had two known SVs, two novel SVs were designated N3 and N4.

### Transcriptome-wide computational framework

We devised an extensive framework to obtain isoform-specific information for all expressed transcripts using Batch CD-Search [17], Mercator [29], RNAfold [30], CPC [18], and CodeWise (Additional file 1: Figure S1). The results obtained from the application of each tool were mined separately and also in conjunction with the other tools to enable functional inference for selected known and novel transcripts. All parameters in the publicly available tools were set to their default values unless otherwise stated. In the following sections, implementation details of each tool are described.

#### Batch translation and Batch-CD Search

First, the in-house Python program BatchTranslator.py (Additional file 2) was used to find the longest protein sequence in each nucleotide sequence in batch mode. This program evaluates all ORFs of a sequence, starting with the AUG start codon and ending with any of the three stop codons, returning only the longest protein sequence. The program produces two separate output files: (i) FASTA protein sequence and (ii) information on translation statistics including the length of the 5’ untranslated region (5’-UTR), potential ORF length, potential ORF ratio (length of potential ORF)/(length of transcript), 5’-UTR ratio (length of 5’-UTR)/(length of transcript), and protein length. The in-house program accepts a FASTA file and returns the most likely ORF of a transcript with accuracies of 99 in *Arabidopsis thaliana*, 96 in *Medicago truncatula*, and 95 % in *Glycine max*, (all data were obtained from Phytozome v9). Second, Batch CD-Search, which accepts up to 100,000 protein sequences at a time, was used to identify conserved domains [17].

#### Mercator

Lohse et al. [29] is a sequence similarity-based functional annotation tool that uses the Basic Local Alignment Search Tool (BLAST) algorithm to identify sequences that resemble the query sequence (above a specified threshold) from several reference sequence databases. Collectively, these databases contain information on all Arabidopsis proteins, proteins from the SwissProt Plant Protein Annotation Program (6,000 plant proteins), 57,000 rice proteins, 17,000 *Chlamydomonas reinhardtii* protein models, and 2,169 domains from InterPro, conserved domain database (CDD), and Eukaryotic Orthologous Groups (KOG) databases. Mercator assigns each transcript to a MapMan ontology bin. The presence of a given transcript in a known MapMan bin helps to predict functionality of that transcript. All parameters were utilized in the Mercator web server and the BLAST cutoff parameter was set to 50.

#### RNAfold and CPC

Available in the Vienna package [30], was used to predict RNA secondary structure and the minimum free energy of all transcripts by using the command-line version of RNAfold in batch mode. The **CPC** web server [18] was used to assess the coding potential of transcripts. CPC uses an SVM classifier trained with respect to sequence similarity (using BLAST) and length (using FrameFinder). Coding potential is predicted with reference to known protein sequences in the UniProt database [31].

### Development of the CodeWise classifier

We developed the CodeWise classifier for accurate assessment of the coding potential of plant transcripts. CodeWise integrates the tools described above with additional features that aid in the categorization of coding versus noncoding transcripts.

#### Features

CodeWise features include: (i) sequence length, potential ORF ratio, UTR ratio, and potential protein length, (ii) sequence content (GC content, and T/A and G/C ratios), (iii) conserved domain information (number of conserved domains and extent of domain truncation), (iv) RNAfold-based minimum free energy of RNA secondary structure, (v) protein sequence similarity and functional annotation (presence of transcripts within the MapMan bins), and (vi) CPC scores. The Batch CD-Search tool was used as described above to identify conserved domains in each amino acid sequence. The extent of domain truncation is reflected in the “truncation ratio” defined as (the number of truncated domains)/(total number of domains).

#### Training and testing

The features described above were compiled for a total set of 115,000 unique *Arabidopsis thaliana* transcripts from The Arabidopsis Information Resource (TAIR) 10 database [32] and *Glycine max* (version 189) transcripts [33], including coding (positive class) and noncoding transcripts (negative class). The LibSVM package was used for implementation of the SVM classifier [34]. The positive training set included 35,000 Arabidopsis transcripts and 50,000 soybean coding transcripts. The negative training set included non-redundant known Arabidopsis noncoding transcripts from 3 resources: (i) 25,000 from the plant long non-coding RNA database PLncDB [19], (ii) 3,800 from the NONCODE version 4 database [35], and (iii) 278 from TAIR10 [32]. There is currently no available source for noncoding soybean transcripts. Due to the low number of available noncoding transcripts, weighted SVM training (−wi weight in LibSVM) with a 3 to 1 ratio was used to prevent unbalanced training. 75 % of the data were randomly selected for training, and the remainder of the data were used for testing, keeping the existing 3 to 1 ratio between the coding and noncoding transcript classes. The training and testing samples were normalized between −1 and +1 prior to training using the *svm*-*scale* program available in LibSVM. Several kernels (radial basis functional (RBF), polynomial, and linear) were used to select the best model. The linear kernel showed the best accuracy of 96 %, followed by the RBF kernel (94 %). Accuracy was determined as the ratio of correct predictions to the total number of transcripts. SAS JMP Pro 11 was used for feature assessment in CodeWise using Linear Discriminant Analysis (LDA) and Principal Component Analysis (PCA).

### Clustering and correlation analyses

GeneCluster 3.0 [36] was used for centering, normalizing, and clustering of SVs into 5, 10, 15, 25, and 30 clusters based on their FPKM values with 500 iterations using the k-means algorithm. Distinct expression patterns within the transcript population were detected in 25 clusters (Additional file 3: Figure S2). Therefore, k = 25 was selected for further visualization in Java Tree View [37] and for data mining. Pearson correlation analysis of sense and antisense transcripts was calculated using an in-house Python program. Sense and antisense transcripts that showed significant correlation of expression over the time course of soybean embryo development were identified (*p*-value < 0.05).

### Co-expression network analysis

ArrayMining was used to construct a co-expression network for the set of 2,938 differentially expressed and alternatively spliced transcripts. ArrayMining yields a weighted gene co-expression network of significantly correlated genes that have similar expression patterns within a user-defined threshold [38]. The Fruchterman-Reingold method was used for network visualization, the edge-adjacency threshold was set to 0.9, and the resulting network was visualized using an organic layout in Cytoscape 3.1 [39]. We define two nodes as nearest neighbors in a network if there is a direct edge connecting those two nodes. If a node (*x*) is connected to *m* nodes and *n* is the total number of nodes in a super-cluster *sc*, the degree of connectivity of *x* in *sc* can be defined as:

$$ Degree\  of\  connectivit{y}_{x,\ sc}=\raisebox{1ex}{$m$}\!\left/ \!\raisebox{-1ex}{$n$}\right. $$

The degree of connectivity for super-cluster *sc* did not follow a normal distribution and median was chosen to represent this distribution. The degree of connectivity for a super-cluster *sc* was defined as:

$$ Degree\  of\  connectivit{y}_{sc} = median\ \left( Degree\  of\  connectivit{y}_{all\  nodes,sc}\right) $$

### Signaling pathway visualization

The in-house tool Beacon was used for the visualization of signaling pathways [40]. The Beacon Pathway Editor consists of a tool designed to draw pathways encoded in the Systems Biology Graphical Notation Activity Flow language that is a standard for describing pathways in terms of perturbations, influences, activities, logical operators, and phenotypes [41].

### Quantitative real-time PCR

Quantitative Polymerase Chain Reaction (qPCR) was performed on selected sense and antisense transcripts, including LEC1-Like (L1L), two ETHYLENE RESPONSE FACTOR/APETALA 2 (ERF/AP2) TFs, gibberellin 2 (GA2) oxidase, and phytochrome-interacting basic helix-loop-helix 5 (PIL5), using samples from several time points to further validate the changes in transcript levels obtained from RNA-Seq as described [25]. The validated transcripts and their specific primers are summarized in Additional file 4: Table S1. Comparison between expression results from RNA-Seq and qPCR are shown in Additional file 5: Figure S3.

## Results

### Overview of the transcriptome-wide computational framework

The data used in this study were taken from an existing transcriptomics data set pertaining to seed filling and early desiccation stages of soybean embryo development [26]. Differential expression analysis of this dataset yielded 17,181 transcripts (many of which were previously unidentified) that showed significant changes in their levels over time (FDR < 0.05) [25]. Some of the newly identified transcripts were novel SVs, intergenic, and/or antisense and of different coding potentials. These types of transcripts, although important in regulating various aspects of cell development [8–10, 12], have been largely neglected in analyses of transcriptomics studies to date.

**Fig. 1:**
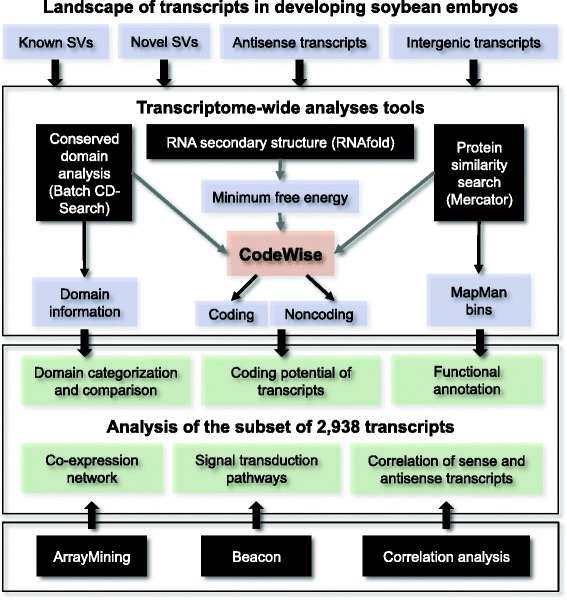
Computational framework. Transcriptome-wide analysis tools comprised large-scale conserved domain search using Batch CD-Search, RNA secondary structure prediction using RNAfold, functional annotation by Mercator, and coding potential assessment using CodeWise. These tools, in conjunction with co-expression network analysis by ArrayMining and signal transduction pathway analysis in Beacon, were used for detailed data analyses of a set of 2,938 transcripts. This population afforded the opportunity to identify candidate SVs transcribed from the same gene with potentially distinct functions in different stages of soybean embryo development. Each of the 1,395 genes had more than one transcript significantly differentially expressed during time-course of soybean embryo development (FDR < 0.05), leading to the detection of 2,938 transcripts. Tools are shown in black, inputs in blue, and outputs in green

Our framework (Fig. 1) involves new and existing tools that were applied (i) globally to all identified known and novel transcripts and (ii) to a set of 2,938 transcripts originating from 1,393 genes. Each of these genes was defined as having at least two significantly differentially expressed SVs in developing soybean embryos. These 2,938 transcripts do not include transcripts that showed stable, non-changing expression levels. While the entire analysis was performed at the transcriptome-wide level, detailed mining of splicing events and function predictions was only performed on the smaller data set of 2,938 transcripts.

The first steps in the analysis included (i) large-scale functional domain analysis by batch CD-Search [17], (ii) predictions of RNA secondary structures and their minimum free energy by RNAfold [30], and (iii) functional predictions and annotations by Mercator [29]. These tools were used independently and also in conjunction with the in-house SVM classifier CodeWise to assess the coding potential of transcripts. Second, additional tools were applied to the set of 2,938 transcripts, including (i) co-expression network analyses by ArrayMining [38] and visualization in Cytoscape [39], (ii) the depiction of inferred signal transduction pathways in the Beacon Pathway Editor [40] based on prior knowledge combined with our data, and (iii) Pearson correlation analysis of sense and antisense transcripts by an in-house python program, all in the context of AS, and timing and patterns of transcript expression. In the following sections, different modules of this framework are explained in detail.

### Transcriptome-wide domain analysis of protein variants

Detection of the presence or absence of functional domains can aid in predicting interactions and therefore, functions of protein isoforms. For example, a novel protein isoform possessing a new domain known to facilitate interactions with signaling proteins of known function can be inferred to potentially interact with these other proteins and function in those signal transduction pathways. Hence, there was a need to obtain global information concerning the presence, absence, and/or truncation of functional domains. All known and novel expressed transcripts were translated *in silico* to identify the longest amino acid sequence and then subjected to Batch CD-Search [17] to identify conserved domains in the set of protein sequences. This transcriptome-wide domain analysis led to the extraction of specific domain information for all expressed transcripts (Additional file 6: Table S2).

### Transcriptome-wide analysis of transcript coding potentials

#### CodeWise classifier development

An important and challenging step for functional predictions is determining the coding potential of transcripts, which is a measure of how likely a transcript is to encode a protein. Noncoding transcripts can potentially interfere with, or otherwise affect, gene expression, which makes them candidates as important transcriptional and post-transcriptional regulators [6, 7, 10, 11]. Integration of as many features as possible improves the accuracy of coding potential prediction tools. We gathered a large compendium of data related to sequence, RNA structure, conserved domains, sequence similarity, and functional annotation of Arabidopsis and soybean transcripts. We used binary SVM classification, which is a supervised learning approach known to yield high accuracy in high dimensional input data such as genomics data [42, 43]. A large spectrum of features was selected for evaluating the coding potential of each transcript in CodeWise to distinguish coding from ncRNAs: (i) sequence length, (ii) sequence content, (iii) presence and truncation of conserved domains, (iv) free energy of RNA secondary structure, (v) protein sequence similarity, and (vi) CPC score.

These features were selected based on the current state of knowledge about the characteristics of coding and noncoding transcripts. First, features related to the nucleotide and protein sequence length were shown to be necessary, but insufficient, for the separation of coding from noncoding transcripts [12, 23, 24]. These features include ORF and 5’-UTR ratios and potential protein lengths. Second, noncoding transcripts were shown to have higher GC content and T/A ratio than protein-coding transcripts [12, 44]. Third, protein-coding transcripts are likely to have conserved domains. Truncation of domains in either the C- or the N-terminus can affect protein function. To obtain information on the presence, absence, and/or truncation of functional domains, transcripts (including ncRNAs) were subjected to computational batch translation and batch CD-search. In the case of ncRNAs, putative start and stop codons and potential peptides can still be identified computationally. Fourth, protein-coding transcripts have more stable secondary RNA structures than noncoding transcripts, which is reflected in their minimum free energy [44, 45]. This parameter was predicted by using RNAfold [30] for all coding and noncoding transcripts. Fifth, protein sequence similarity and functional annotation can be important to distinguish coding from noncoding transcripts. Mercator [29] was used to assign transcripts into MapMan [46] ontology bins based on protein sequence similarities. Sixth, CPC is used to assess the coding potential of transcripts [18]. Incorporation of the CPC score as a feature in CodeWise was evaluated as well. These features were tested together and in different combinations to assess their importance for the overall accuracy of CodeWise.

#### CodeWise performance evaluation

**Fig. 2:**
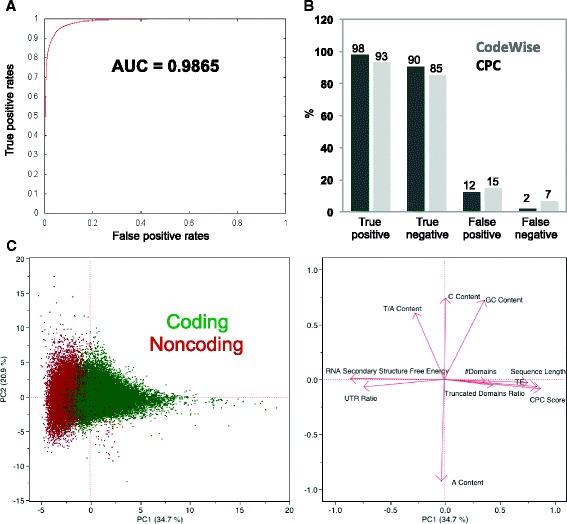
CodeWise performance evaluation. **a** ROC curve on the test set. **b** Comparison of CodeWise and CPC prediction power on the same set of known coding and noncoding transcripts. True positive: a coding transcript correctly predicted to be coding, true negative: a noncoding transcript correctly predicted to be noncoding, false positive: a noncoding transcript incorrectly classified as coding, false negative: a coding transcript incorrectly classified as noncoding **c** PCA results showing correlations of all features used to train the classifier. Score and loading plots for the first two principal components are shown on the left and right, respectively. Eigenvectors are shown as red arrows in the loading plot

CodeWise classified transcripts into coding and noncoding groups with the area under the receiver operating characteristic curve (AUC) > 0.98 on an independent test set, when all features were used for training (Fig. 2a). For assessing the coding potential of transcripts in CodeWise, no predetermined cutoff was used for distinguishing coding from noncoding transcripts with respect to protein length and sequence similarity. Instead, the classifier learned the cutoffs and patterns that exist between coding and noncoding classes among the training features. CodeWise assigned both coding and noncoding probabilities to each transcript. CodeWise outperformed CPC by a higher number of true predictions and a lower number of the false predictions (Fig. 2b). Because the other available tools, specifically iSeeRNA, PhyloCSF, and CPAT do not include plants as a model system, evaluating their performance relative to CodeWise was irrelevant.

We used three methods to assess the contribution of different features within CodeWise. First, the CodeWise classifier was trained and tested with all combinations of six feature groups (127 combinations). Second, principal component analysis (PCA) was performed to evaluate how different features contributed to the variance between coding and noncoding classes (Fig. 2c). PCA results revealed that sufficient separation (with only a small proportion of outliers) between the coding and noncoding transcripts was achieved solely through principal component 1 (PC1), which accounted for 34.3 % of the variance. PCA also revealed the positive and negative correlations among the specific individual features (depicted as eigenvectors aligning in the same and opposite directions, respectively, along the PC1 axis in the loading plot of Fig. 2c). Third, LDA was used to find linear combination of features with the highest covariate scores for separation of coding and noncoding transcripts. LDA resulted in 93.94 % correct classification of coding and noncoding transcripts with AUC of 0.9797 for both coding and noncoding classes. These three evaluation techniques revealed that at least three specific feature groups are required for separation of coding from noncoding transcripts: (i) the free energy associated with RNA secondary structure, (ii) the presence of conserved domains, and (iii) sequence features (5’-UTR ratio, potential ORF ratio, and protein length). Because CPC scores are highly correlated with ORF ratio and protein length, this feature does not affect CodeWise predictions (Fig. 2b). No significant differences were observed in the nucleotide content, specifically between the GC content or the T/A ratio, of coding transcripts and noncoding transcripts (Fig. 2b).

**Fig. 3:**
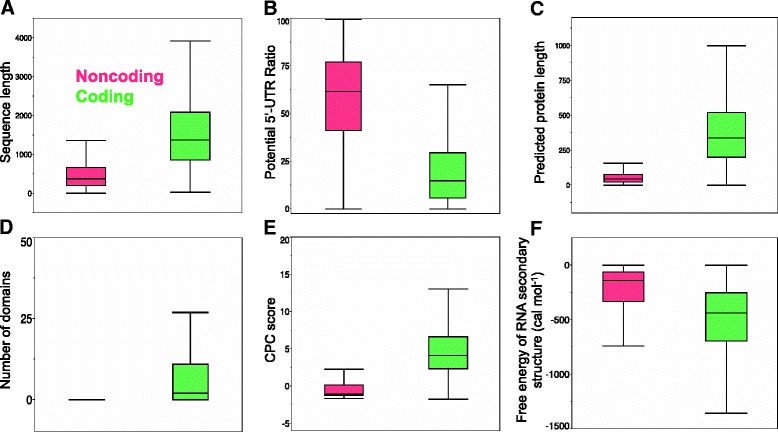
Box-and-whisker plot comparisons of coding and noncoding transcripts predicted by CodeWise with respect to individual features. **a** Sequence length. **b** Potential 5′-UTR ratio. **c** Potential protein length. **d** Number of conserved domains. **e** CPC score. **f** Minimum free energy of RNA secondary structure

Noncoding transcripts had significantly higher minimum free energy of RNA secondary structure and tended to be shorter, with higher 5’-UTR ratio, lower potential ORF ratio, shorter predicted protein lengths, no conserved domains, and lower CPC scores than coding transcripts (Fig. 3). The average minimum free energy of RNA secondary structure of coding and noncoding sequences was −371 and −134 cal mol^−1^, respectively (Fig. 3f).

#### Transcriptome-wide analysis of coding and noncoding transcripts in developing soybean embryos using CodeWise

Transcriptomics analysis revealed the expression of 39,101 known and 64,005 novel transcripts in developing soybean embryos (Table 2). The transcript population included transcripts from genic and intergenic regions. Based on the Cuffdiff2 analysis, 17,181 out of 103,106 transcripts showed significant differential expression. On average, a soybean gene produced three transcripts and, for the most part, the previously known SVs showed significantly higher expression than the novel transcripts (*p* < 0.0001, *t*-test). Known transcripts had significantly higher average FPKM values than antisense, intergenic, novel genic sense, and overlapped transcripts (*p* < 0.0001, *t*-test). Using the CodeWise classifier, we identified 13,652 lncRNAs, including 10,023 genic lncRNA, 1,064 lincRNAs, and 2,295 noncoding antisense transcripts (Additional file 7). Based on CodeWise test results on coding and known noncoding plant transcripts compiled from existing databases [19, 32, 33, 35], we estimated that about 96 % (AUC > 0.98) of these lncRNAs were correctly rejected as coding (true negatives). Long noncoding transcripts showed significantly lower expression than coding transcripts (*p* < 0.0001, *t*-test), which is consistent with other studies [23, 47, 48].

**Table 2:** Transcript distribution among different classes of significantly changed transcripts

Transcript classes	Transcript number	Significantly changed
Known (=)	39,101	13,398
Novel splice junction (j)	57,376	2,840
Overlapped (o)	1,689	252
Antisense exon (x)	2,266	242
Antisense intron (s)	599	30
Intergenic	2,075	419
Total	103,106	17,181

Cuffdiff2 was used for time-course differential expression analysis at isoform level. Transcripts that were differentially expressed at least at one time point compared with previous time point with FDR < 0.05 were defined as significantly differentially expressed. Out of 103,106 transcripts detected in developing soybean embryos, 17,181 transcripts were significantly changed

## Bioinformatics analyses of AS events

### Identification of alternatively spliced and significantly differentially expressed transcripts

We previously identified 1,393 genes, each with more than one significantly differentially expressed transcript, resulting in a population of 2,938 known and novel SVs and antisense transcripts [25]. This population afforded the opportunity to identify candidate SVs transcribed from the same gene with potentially distinct functions in different stages of soybean embryo development. Transcriptome-wide analysis of this relatively small subset revealed several interesting phenomena in the context of embryo development. For example, this dataset includes (i) SVs with different splicing patterns covering major developmental stages of developing soybean embryos, (ii) coding and noncoding transcripts such as lncRNAs and antisense transcripts, (iii) SVs with different number and types of conserved domains with the same and/or different expression profiles.

**Fig. 4:**
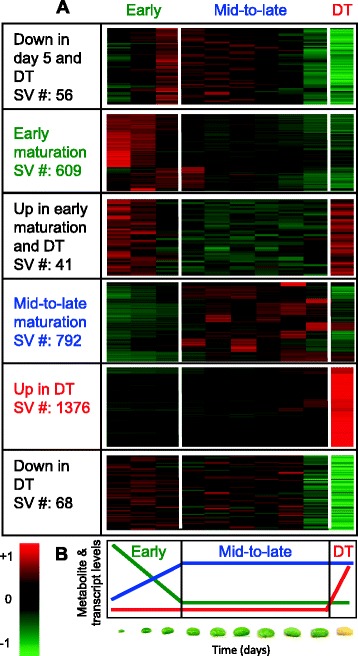
Assembly of super-clusters. **a** Normalized FPKM values of the set of 2,938 transcripts were clustered into 25 groups using the k-means algorithm. Clusters with similar expression profiles across developmental stages were grouped into six super-clusters. Three major developmental stages (early maturation (green), mid-to-late maturation (blue), and desiccation (red)) containing 94.5 % of the transcript population were identified. **b** Trends involving changes in metabolites and transcript levels [28] were grouped by developmental stages and color coded as corresponding three major super-clusters

To illustrate the use of our framework for biological data mining and function inference, this set of transcripts was further analyzed in several ways. The k-means clustering algorithm was used to group these 2,938 transcripts into 25 clusters (Additional file 3: Figure S2) that represented major trends in seed filling and early desiccation-related processes. Transcripts belonging to the individual clusters are presented in Additional file 8: Table S3. Some of the clusters displayed similar trends and were therefore merged into six super-clusters (Fig. 4a), based on prior knowledge obtained from the same dataset concerning the timing of metabolite and metabolism-related transcript accumulation [26]. Three basic trends reflecting changes in metabolite and transcript levels (Fig. 4b) included: (i) early maturation - initial decrease until day 15 – 20, followed by stable low levels (green trend), (ii) mid-to-late maturation - initial increase, followed by stable high levels (blue trend), and (iii) desiccation (DT) - appearance of metabolites and transcripts in yellow embryos at day 55 (red trend). Overall, the transcripts were not evenly distributed among the six super-clusters. The majority of AS events were observed in the DT super-cluster, followed by the early and mid-to-late super-clusters (Additional file 9: Figure S4). Known and novel splice junction SVs dominated all super-clusters, but a small number of transcripts belonging to other classes (exon skipping and antisense) were also observed in nearly all super-clusters (Additional file 9: Figure S4).

The three temporal patterns provided the basis for further mining of the population of 2,938 SVs, which included (i) expression patterns, (ii) presence or absence of conserved domains, (iii) functional annotation based on protein similarity by Mercator, (iv) CPC- and CodeWise-derived coding potential predictions (Additional file 8: Table S3), (v) potential ORF ratio and 5’-UTR length, and (vi) GC content. Classification of 2,605 transcripts was consistent between CodeWise and CPC, while 333 transcripts were reclassified by CodeWise. Based on the testing results presented above, 96 % of these predictions are estimated to be correct.

### Conserved domain analysis of potential protein variants

**Fig. 5:**
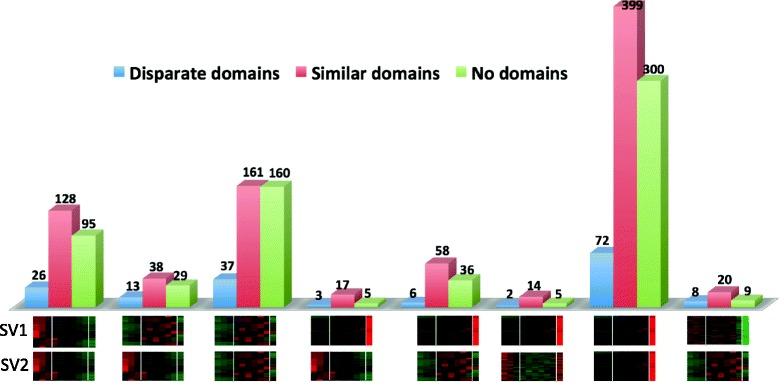
Pairwise comparisons of transcripts with respect to their domains and expression patterns across the population of 2,938 transcripts. Batch CD-Search results were mined to identify transcripts encoding proteins and peptides with disparate, similar, or no known domains. Each gene in this set had at least two transcripts that were differentially expressed. Thus, transcripts originating from a gene may belong to the same (hence same super-cluster comparisons) or different super-clusters (hence different super-cluster comparisons). SV pairs belonging to the same or different super-cluster pairs are shown below for each comparison

We define an “SV group” as those isoforms in the population of 2,938 transcripts that were spliced from the same pre-mRNA. Members of each SV group were divided into three categories in terms of differences in their conserved domains. This categorization was done by performing pairwise domain comparisons of isoforms within each SV group on Batch-CD Search results (Fig. 5), with the focus on (i) disparate domains, defined as SVs differing in at least one conserved domain, (ii) similar domains, defined as SVs having the same types of domains, but the number of domains can be different, and (iii) no known domains, defined as at least one of the SVs lacked any conserved domains. This domain categorization allowed the exploration of differences among SVs with respect both to their functional domains and timing of expression, which can facilitate prediction of possible functional roles of different SV pairs. SVs having different expression profiles (reflected in their presence in different super-clusters) with different number and types of conserved domains may play distinct roles in developing embryos. Domain comparisons among SVs present within the same super-cluster were also performed to obtain information about SVs that had similar expression profiles.

Interestingly, the majority of SVs (80 %) originating from the same gene co-expressed and belonged to the same super-cluster. Others were expressed at different times (different super-clusters, e.g., “DT, early maturation”). The majority of SV groups either had similar domains (48 %) or one of the SVs in the group lacked any known domain, regardless of super-cluster comparisons (37 %) (Fig. 5). The group of SVs with no domains included both sense lncRNAs (12 %) and transcripts encoding peptides or proteins with no known domains (14 %). For example, 777 SV pairs were up-regulated at the DT stage (both SVs belonged to the DT super-cluster). Among these SV pairs, the majority had similar domains (399 SV pairs), while only 72 SV pairs had disparate domains. The remainder of the SV pairs contained one partner SV with no known domain. Eighty SV pairs belonged to the mid-to-late and early maturation super-clusters. While these SV pairs had completely different expression profiles, 38 and 42 SVs had similar and different, respectively, conserved domains.

### Sense and antisense transcript pair analysis

Emerging studies provide evidence that natural antisense transcripts play an important role in regulating gene expression [49, 50]. RNA-Seq analysis enabled the identification of 167 novel sense and antisense transcript pairs that showed changes in expression during soybean embryo development. A plausible hypothesis is that if a corresponding sense and antisense transcript pair shows positively or negatively correlating expression patterns, then the stability of the sense transcript will be affected by the antisense transcript. For sense and antisense transcript pairs, potential correlations were investigated using Pearson correlation analysis. The majority of sense-antisense pairs (155 out of 167 pairs) had significantly correlated expression profiles during soybean embryo development (Additional file 10: Table S4). Specific examples of potential antisense regulation will be discussed in detail in section 4.4. in relation to ABA and/or FUS3 action and timing of their expression.

### AS events related to ABA and/or FUS3 action

FUS3 plays a key role in the regulation of seed development [51], as does the phytohormone ABA [1]. It was therefore of great interest to understand the relationship of RNA splicing and antisense regulation to ABA- and FUS3-related events in developing soybean embryos and to search for possible clues to as yet unknown and/or partially understood regulatory mechanisms. Therefore, we mined the set of the 2,938 transcripts for potential ABA- and FUS3-related targets. ABA-related Arabidopsis genes were extracted from [1] and included proteins involved in ABA metabolism and signaling, as well as those associated with interactions of ABA with other hormone-mediated pathways. Similarly, the identity of the genes in the FUS3 regulon in Arabidopsis was obtained from [51]. The Arabidopsis gene IDs associated with the corresponding soybean genes encoding these 2,938 differentially expressed transcripts were cross-referenced with the Arabidopsis ABA-related and FUS3-regulated genes to obtain ABA-related and FUS3-regulated potential homologs in soybean. This mining led to the detection of 318 transcripts (Additional file 11: Table S5A). These transcripts were carefully examined with respect to conserved domains, coding potential, and functional annotation. The majority of ABA-related transcripts were expressed during the mid-to-late and DT phases of soybean embryo development (89 %). FUS3 is encoded by two genes in soybean, each producing one transcript (Glyma16g05480.2 and Glyma19g27336.1) in developing embryos, both genes showing similar and stable expression until day 55 when their levels dropped significantly [26]. The FUS3 regulon [51] contained 181 transcripts, some of which are also related to ABA signaling, showed differential expression during soybean embryo development.

### AS events related to ABA and/or FUS3 action during early maturation

The early super-cluster is relevant to young, fully differentiated, green embryos that expressed genes associated with various aspects of cell division but already had started to accumulate seed storage compounds [26]. The FUS3-related SVs belonging to the early super-cluster included: (i) L1L, (ii) receptor protein kinase barely any meristem (BAM) 2 and calcium-dependent protein kinase (CPK) 11, and (iii) a component of 26S proteasome-mediated protein degradation radiation sensitive (RAD) 23. A number of Auxin response factors (ARFs) and regulatory proteins involved in flower development-related cell division and differentiation, some of which are connected to regulating seed development [52–54], were also identified.

**Fig. 6:**
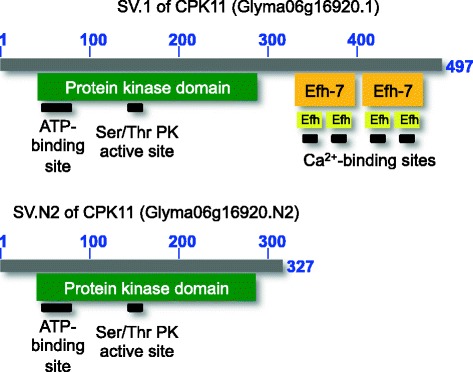
Graphical representations of functional domains present in known (Glyma06g16920.1) and novel (Glyma06g16920.N2) CPK11 protein isoforms. Resulting protein variants are shown in gray with length intervals and the actual lengths indicated by blue numbers. The corresponding domains are shown below each protein variant. Active and molecule-binding sites are shown below the relevant functional domains. The sizes and arrangements of these domains and sites reflect the reality. The novel CPK11 protein variant retained the protein kinase domain, but lost the EF-hand Ca^2+^-binding domain. Efh, EF-hand domain; PK, protein kinase

Some soybean SVs that were expressed during the mid-to-late phase showed differences with respect to their respective functional domains (ARF2 and 6, CPK11, and RAD23). For example, a novel CPK11 SV was missing the EF-hand (EFh) domain present in the canonical SV (Fig. 6). The novel RAD23 SV lacked a ubiquitin (UBQ) superfamily domain. AS can also change the protein sequence, so that the SVs resemble different, but related proteins, which was observed for ARF6 and 8 (Glyma02g45100.N2 and 1). Interestingly, the novel ARF6 SV also lacked two domains, but had a new PB1 superfamily domain found in dimer-forming protein kinases [55–57].

#### AS events related to ABA and/or FUS3 action during mid-to-late maturation

The mid-to-late super-clusters included SVs that showed increased transcript levels during the stages of steady-state seed storage compound accumulation [26]. Only three alternatively spliced FUS3 targets connected to the B3 network, belonging to the mid-to-late super-cluster were identified: (i) the transcriptional regulators HAP2A nuclear factor YA (NF-YA) 1 and bZIP66 (ABA-responsive element binding protein AREB3) and (ii) the E3 UBQ ligase DREB2A-interacting protein (DRIP) 2. While the HAP2A protein was not affected by AS, bZIP66 Glyma03g00580.N2 had ten additional amino acid residues at the C terminus and an extended 5’-UTR. The DRIP2 SV Glyma02g15980.N2 lacked the C3HC4-type Really Interesting Gene (RING) finger domain important for protein-protein interactions of UBQ ligases [58] and belonged to the early super-cluster.

#### AS events related to ABA and/or FUS3 action during DT

The DT super-cluster contains predominantly ABA-related SVs that showed basal transcript levels during all seed filling phases and high expression in yellow embryos at day 55 [26] (Additional file 12: Figure S5A). ABA-related SVs included transcripts encoding proteins similar to: (i) the epigenetic regulator histone deacetylase (HDAC) 6 associated with chromatin remodeling, (ii) two regulatory components of ABA- and G-protein related receptors REGULATORY COMPONENTS OF ABA RECEPTOR 3 (RCAR3) and G-PROTEIN COUPLED RECEPTOR 1 (GCR1), respectively, (iii) the transcriptional regulators of ABA signaling no apical meristem (NAM) TF (ATAF1) and ABI5-binding proteins (AFPs), (iv) sucrose nonfermenting related kinase protein (SnRK2.6), and (v) signaling-related phospholipase D delta (PLDdelta). ATAF1 was also identified in the FUS3 regulon. The only other FUS3-regulated SVs related to seed maturation and expressed in DT were those encoding saposin-like Asp proteases.

Differences with respect to protein length, number of domains (e.g., PLD delta, ATAF1, saposin-like Asp proteases), the absence of any known domains, (AFP4), and length of either the 5’ or the 3’ UTR (GCR1) occurred among different SV pairs. Variants of the same protein that showed similarity to different, but related, Arabidopsis proteins were also identified. While the SV Glyma04g38560.1 was similar to ATAF1 (At1g01720), Glyma04g38560.N2 was more related to At5g63790, a NAM domain-containing protein (NAC) 102, than to ATAF1. Similarly, Glyma17g01500.1 was similar to the saposin-like Asp protease At1g62290, but N3 resembled a different vacuolar protease (At1g11910). Interestingly, AS did not affect the structure of the G protein-coupled receptor domain in the novel, slightly shorter GCR1 protein, instead, the two SVs differed with respect to their 3’-UTRs. Differential expression was also observed in other SV pairs. The novel PLDdelta and HDAC6 SVs belonged to the early super-cluster, and the novel GCR1 SV to the mid-to-late super-cluster.

#### Antisense events related to ABA and/or FUS3 action

SV groups of ABA- and FUS3-related genes that were co-expressed during the same developmental phase showed multiple differences. AS lead to the production of lncRNAs and/or differences in protein sequence, number and types of functional domains, in 5’- and 3’-UTR length and sequence, and expression patterns. While these changes were detected in all phases of soybean embryo development, the occurrence of antisense transcripts among the ABA- and/or FUS3-related transcripts was confined to the early and DT phases. Antisense transcripts expressed at the early phase include those associated with genes encoding ABA glucosylase, L1L, BAM2, and genome-uncoupled (GUN5) (a putative ABA receptor at the chloroplast envelope) [59, 60]. The only exception was an antisense transcript associated with a putative cytokinin transporter *PUP1* gene. This antisense transcript co-expressed with its sense transcript during the mid-to-late phases.

Several antisense transcripts were also detected at day 55 of embryo development (DT) and appear to be connected to processes involving interactions of ABA with other phytohormones. Overall, 23 transcript pairs, regardless of any relation to ABA signaling, in which one member of each pair was antisense, together with seven single antisense transcripts without an accompanying sense transcript were detected at day 55 (Additional file 11: Table S5B). Among this population, several transcripts encoding proteins related to GA or ethylene signaling were present in both antisense and sense orientations and included GA2 oxidase, several ERFs, and PIL5 protein.

### Generation and analysis of co-expression network

**Fig. 7:**
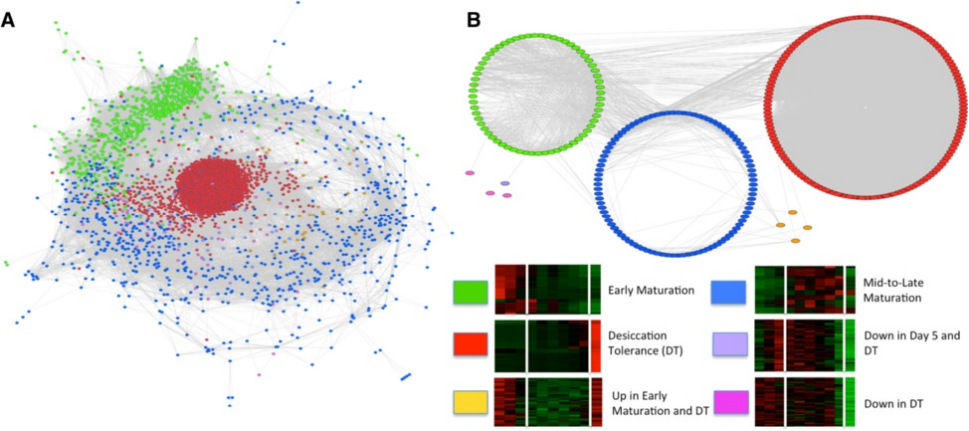
Isoform-specific co-expression network for the set of 2,938 transcripts. ArrayMining was used to construct the co-expression network. The resulting network was visualized in Cytoscape. Transcripts (nodes) are color-coded based on their respective super-cluster. **a** Co-expression network visualization of the 2,938 transcripts in the organic layout. **b** Co-expression sub-network for all 318 ABA-related and FUS3-regulated targets (see Results AS events related to ABA and/or FUS3 action section) grouped by super-cluster. The relative density of edges (in gray) reflects different degrees of connectivity among super-clusters. The majority of ABA- and FUS3-related transcripts (310) were located in the three major super-clusters

Co-expression networks have been used to infer potential gene interactions and functions [61, 62]. However, the majority of these networks have been limited to genes due to lack of isoform-specific transcript information. Here, ArrayMining [38] was used to obtain an isoform-specific co-expression network for the set of 2,938 transcripts (Fig. 7a). In the resulting network, each node represents a transcript and is colored according to its respective super-cluster. To reveal possible specific relationships among transcripts belonging to ABA- and FUS3-related events, the 318 transcripts (see AS events related to ABA and/or FUS3 action section) were identified within the co-expression network (Additional file 11: Table S5A). These ABA- and/or FUS3-related transcripts were used to generate a sub-network, reflecting temporal expression in the context of six super-clusters (Fig. 7b). Of the 318 total transcripts encoded by target genes associated with FUS3- and/or ABA–related function, 311 transcripts were located within the three super-clusters corresponding to the three major phases of soybean embryo development.

#### Identification of the hubs

**Fig. 8:**
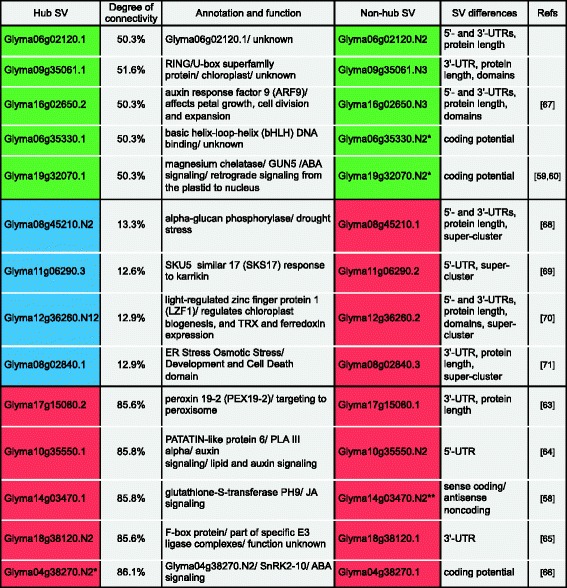
Hubs associated with the three major super-clusters. An in-house python program was used to identify the most highly connected nodes (transcripts) within each super-cluster. The corresponding non-hub SV is also included in the table. Structural or functional differences among these SV-pairs are also indicated. Super-clusters are color-coded as follows: green for early maturation, blue for mid-to-late maturation, and red for DT. Degree of connectivity reflects how well transcripts are connected to each hub (number of connections/ total number of transcripts). *lncRNA, **antisense lncRNA

Although the co-expression network separates different super-clusters, interpretation of connections among the nodes remained intractable due to the large size of this network. To address this problem, we identified the most highly connected nodes (hubs) within each super-cluster. Hubs are the key network properties that reduce network complexity to the major connectors. In all cases, only one transcript derived from the same soybean gene was found to be a hub. Available functional information for hubs is presented in Fig. 8. Approximately 50, 13, and 86 % of transcripts were significantly connected to the hubs represented by transcripts of diverse functions in the early, mid-to-late, and DT super-clusters, respectively. Five hubs with a large number of associated nodes were identified in the case of DT. Among the DT-associated hubs were transcripts encoding soybean proteins similar to Arabidopsis peroxin 19 targeted to the peroxisome [63], PATATIN-like protein 6 involved in lipid and auxin signaling [64], redox-related GST PH9 protein implicated in JA signaling [58], an F-box protein associated with an E3 UBQ ligase complex [65], and a lncRNA transcribed from a homolog of At1g60940, SnRK 2.10 involved in ABA signaling [66].

#### Identification of the nearest neighbors of GCR1 and CPK11

GCR1 and CPK11 are two putative regulators of seed development belonging to the group of 318 ABA- and/or FUS3-related transcripts. *GCR1* and *CPK11* pre-mRNAs were alternatively spliced in developing soybean embryos and the resulting SVs were present in different super-clusters. The domain composition of each GCR1 and CPK11 SVs was confirmed by using the InterPro database [16]. In Arabidopsis, GCR1 (At1g48270) is an ABA-responsive, G-protein-related receptor component distinct from the well-studied RCAR group of receptors [67]. CPK11 (At1g35670) is a protein kinase acting as a positive regulator of ABA/FUS3-mediated responses during seed filling [68]. Guilt-by-association of GCR1 and CPK11 SVs with transcripts of known functions can yield improved understanding of their regulation and function.

**Fig. 9:**
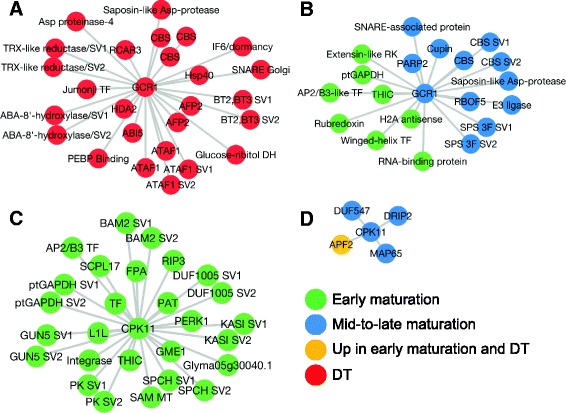
Sub-networks showing the nearest neighbors of the known and novel GCR1 and CPK11 SVs. **a** Known GCR1, **b** novel GCR1, **c** known CPK11, and **d** novel CPK11. The sub-networks were generated by extracting the first neighbors of these four transcripts from ArrayMining results shown in Fig. 7. Nodes are color-coded based on their corresponding super-cluster. Detailed information on annotation, domains, coding potentials, and timing of expression is available in Additional file 13: Table S6

To further elucidate isoform-specific functions of these two important regulators, the nearest neighbors of GCR1 and CPK11 were identified in the corresponding sub-networks (Fig. 9, Additional file 13: Table S6) originating from the ABA/FUS3-related co-expression network (Fig. 7b). The two GCR1 SVs were expressed at the DT and mid-to-late stages, respectively, and were associated with two distinct groups of transcripts. The nearest neighbor group comprising 38 nodes representing SVs expressed during DT was associated with Glyma17g33480.1-encoded GCR1 (Additional file 13: Table S6A). Transcripts associated with Glyma17g33480.N3 were differentially expressed during the early and mid-to-late phases of embryo development (Additional file 13: Table S6B).

The nearest neighbors belonging to the DT-based sub-network were primarily related to signaling or defense. Three coding and one noncoding ATAF1 transcripts were also among the nearest neighbors of GCR1, as were lncRNAs of heat shock protein (Hsp40) and BT2/ BT3. Only two lncRNAs of unknown function were identified as the nearest neighbors of the novel GCR1. A number of coding transcripts were also identified as strongly co-expressing with either the known or novel GCR1 SV. These transcripts encoded enzymes involved in seed filling or germination-related metabolism, including glucose/ribitol dehydrogenase, saposin-like Asp proteases, thiamine biosynthesis protein C (THIC), plastidic glyceraldehyde-3-phosphate dehydrogenase A (GAPDH), and sucrose-phosphate synthase. Homologs of regulatory proteins, including a histone deacetylase (HDAC), Hsp40, and several SVs encoding cystathionine beta-synthase (CBS)-domain-containing proteins that are associated with SnRK1 and energy sensing [69, 70] were also identified. Thioredoxin, rubredoxin, and nicotinamide adenine dinucleotide phosphate (NADPH oxidase, RBOF5) represented the redox-related proteins. It is notable that many SVs present in the sub-networks with the centered GCR1 SVs were “singletons”, that is to say that only one member of a given SV group was present as a node in the sub-network. Interestingly, counterparts of some of these singletons associated with the known CPK11 SV and also belonged to the FUS3 regulon (GAPDH, THIC, saposin-like Asp proteases, and AP2/B3-like TF).

The full length CPK11 (Glyma06g16920.1) strongly co-expressed with several ABI3- and/or FUS3-regulated genes encoding alternatively spliced regulatory proteins (L1L, GUN5) (Fig. 7b). Both BAM2 SVs and a Ser carboxypeptidase-like protease (SCPL17) were identified as the nearest neighbors of CPK11 that also belonged to the FUS3 regulon. The novel SVs of S-adenosyl-L-methionine methyltransferase (SAM MT), pyruvate kinase, and SPEECHLESS (SPCH) were also identified as the nearest CPK11 neighbors and reclassified by CodeWise as lncRNAs with high probabilities (84, 99, and nearly 100 %). For metabolism-related processes, the corresponding SVs encoded enzymes involved in fatty acid/oil biosynthesis or storage (fatty acid synthase KAS1), amino acid metabolism (Asp/2-oxoglutarate aminotransferase PAT), and ascorbate biosynthesis (guanosine diphosphate (GDP)-mannose-3,5-epimerase 2 GME1) (Additional file 13: Table S6C). The CPK11 SV Glyma06g16920.N2 had only four nearest neighbors, two of which were connected to the B3 network (AFP2 and DRIP2) and the novel AFP2 SV was predicted to be lncRNA with a 95 % probability (Additional file 13: Table S6D).

## Discussion

Current high-throughput transcriptomics data reveal a global occurrence of diverse types of transcriptional and post-transcriptional events, leading to the formation of transcripts of different coding potential and strand orientation. Knowing the coding potential and other characteristics of transcripts, including sequence similarity and presence of functional domains in resulting proteins, represents a first step towards discovering novel functionalities. We developed an integrated computational framework involving (i) a transcriptome-wide analysis of functional domains in proteins and an in-house SVM classifier, CodeWise, that categorizes transcripts as coding and noncoding and (ii) a relatively small-scale network analysis of 2,938 transcripts focusing on temporally driven co-expression and co-regulation.

### Integrating various features improved accuracy of CodeWise predictions

Domain analysis yielded predictions regarding transcript and protein functions that can be experimentally validated. CodeWise enabled classification of known transcripts into coding and noncoding categories with AUC over 0.98 when trained and tested with a comprehensive list of features. In most cases, the decision regarding noncoding classification was straightforward (e.g., transcripts with short putative ORFs, long 5’- and 3’- regions, no conserved domains, and high free energy of RNA secondary structure). The free energy of RNA secondary structure represents an intrinsic feature related to structural stability of transcripts [71] and its inclusion improved classifier performance. Predicting the coding potential of transcripts was challenging only in cases of conflicting results obtained from different features, specifically, concomitant presence of features characteristic for coding and noncoding transcripts. Such transcripts can be identified based on their low coding and noncoding probabilities (e.g., 0.45 and 0.55, respectively). CodeWise had low false positive and negative rates and outperformed CPC in accuracy due to the additional features used.

### Landscape of transcripts in developing soybean embryos

CodeWise was used to classify all transcripts detected in developing soybean embryos and, in combination with other tools, to analyze a set of 2,938 differentially expressed and alternatively spliced transcripts in terms of coding potential, expression timing, and changes in number and types of domains. The time period in seed development examined in this study extended from early maturation through the acquisition of dormancy and DT. Our analyses demonstrated the existence of a changing population of multiple types of transcripts over this part of soybean embryo development.

Interestingly, a relatively high proportion of coding and noncoding transcripts with no known domains were detected overall (27 % of total), especially during DT. Many coding transcripts were predicted to encode small proteins lacking known domains (<120 amino acids; encoded by small ORFs). Considerable conservation of small ORFs across five leguminous species (including soybean) and Arabidopsis has been demonstrated [72], suggesting that these are bona-fide proteins that probably act through conserved mechanisms. Known pathways, including sucrose signaling, in which small ORFs participate as “peptoswitches”, were identified in plants [73]. We have identified instances of transcripts that were classified as coding with no known domains that are associated with ABA signaling (Additional file 11: Table S5A), but their potential function as peptoswitches remains to be investigated.

Long noncoding and antisense transcripts have also been implicated in regulating development and signaling in plants [15, 71, 74]. With respect to coding/long noncoding or sense/antisense transcript pairs, a starting hypothesis is that their co-expression leads to either chromatin modification and/or degradation or stabilization of the sense mRNA [7, 11, 50]. The majority of SVs (80 %) derived from the same gene (SV group) belonged to the same super-cluster, including long noncoding and antisense transcripts and their respective coding partners. The overall significance of these co-expression results is not yet clear, but it could be reflecting important conserved transcriptional and/or post-transcriptional regulatory mechanisms.

### ABA- and FUS3-related transcripts were highly connected within the co-expression network of developing soybean embryos

ArrayMining [38] was used to generate an isoform-specific co-expression regulatory network for the set of 2,938 transcripts (Fig. 7a). The resulting co-expression network showed three different kinds of strong associations among the transcripts present in the different super-clusters (Fig. 7b). First, transcripts from ABA- and FUS3-related regulons were identified within the overall network, revealing a specific sub-network. Transcripts within this sub-network were tightly clustered primarily around the three major super-clusters (early maturation, mid-to-late maturation, and DT). This clustering validated the original arrangement of the data into these temporally-based super-clusters. Second, GCR1 and CPK11 SVs expressed at different phases were found to have mostly distinct nearest neighbors, though some SVs were shared between the GCR1 and CPK11 sub-networks, providing a link between AS-related regulation of ABA- and FUS3-mediated signaling. In the case of the canonical GCR1 SV, the nature of its nearest neighbors may correspond to a specific coordinated regulatory mechanism involving AS, chromatin remodeling, redox-related processes, and signaling during DT. The presence of several lncRNAs among the nearest neighbors of GCR1 suggests that AS events involving the production of these lncRNAs are part of a distinct regulatory mechanism related to GCR1 action. Third, five hubs (including a lncRNA transcribed from a homolog of At1g60940, SnRK 2.10) with a large number of associated nodes were identified computationally in the case of DT, providing a connection between AS, redox regulation, and signaling pathways.

### Evidence for post-transcriptional events leading to coordinated pre-mRNA splicing

Transcripts of some of the best-studied TFs and ABA biosynthetic genes that are known to regulate seed development (ABI3, FUS3, and 9-cis-epoxycarotenoid dioxygenases (NCED) 1, 4, and 5 [1]) were present at relatively high and stable levels throughout soybean embryo maturation. Activities of ABI3 and FUS3 are also regulated through protein phosphorylation [75, 76] and proteosomal degradation [77, 78]. ABA-mediated signaling leads to the induction of specific SnRK kinases activating FUS3 and ABI3 [75, 79, 80]. Phosphorylation of FUS3 increases the stability of these short-lived proteins [76]. However, SnRK1.1 (represented by Glyma08g26180 and Glyma08g26191) transcript levels remained stable during soybean embryo development [26], suggesting that any differential regulation mediated by this kinase would have to be at its translational and/or posttranslational levels.

While FUS3 transcript levels and, possibly, protein activity remained stable in developing soybean embryos, many SV pairs of ABA-related and FUS3-regulated genes were differentially expressed and did not co-express with FUS3. Differential expression of SVs originating from the same pre-RNAs suggests the occurrence of post-transcriptional events, which can globally influence transcript levels and stability. This is consistent with the observation that many ABA-related and FUS3-regulated transcripts that originated from different, but functionally related genes were co-expressed in the data set. It appears that specific splicing components can regulate differential splicing of groups of pre-mRNAs during specific stages of embryo development, leading to differential temporal expression of these SVs. It is tempting to hypothesize that this coordinated splicing (“co-splicing”) may be a common regulatory mechanism employed in signaling processes within the embryo developmental programs. AS was proposed as a global regulatory mechanism in seed dormancy [81], and it also could be the case in developmental transitions within embryo maturation.

#### Potential roles for alternate pathways and antisense regulation in phytohormone interactions during late seed maturation and germination

The majority of ABA-related SVs corresponded to Arabidopsis genes already documented to participate in dormancy or, in some cases, germination. It is also interesting to note that a SV of a homolog of RCAR3/ PYRABACTIN RESISTANCE 1-LIKE (PYL 8) implicated in ABA signaling that promotes dormancy [82] was differentially expressed during DT, whereas an SV corresponding to PYL6 was expressed during the mid-to-late phase. Given the differences in the population of SVs that are ABA-related and were expressed during one or other of the two phases, it is possible that distinct ABA signaling pathways are in operation during the two developmental phases (Additional file 12: Figure S5A).

**Fig. 10:**
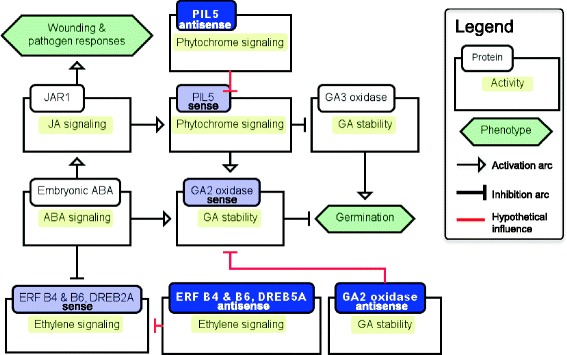
Proposed interactions of ABA with GA, ethylene, and phytochrome signaling pathways involving potential post-transcriptional regulation by antisense transcripts during desiccation phases and/or seed germination. These simplified signaling pathways were drawn in the Beacon editor. Signaling components involving sense-antisense transcript pairs are shown in two different shades of blue. The influence arcs do not imply the direct interactions; they indicate a positive or negative influence between the corresponding glyphs

Transcripts of putative soybean homologs of Arabidopsis genes known to be associated either with ABA-related events (including, but not restricted to signaling), and/or to be targets of FUS3 were associated with specific AS events or antisense expression. AS resulted in altered numbers or types of domains and production of coding and noncoding transcripts, which has consequences for molecular interactions, epigenetic events, regulation of protein activity, and subsequently function. This information was incorporated into proposed signaling pathways using the Beacon editor (Fig. 10, Additional file 12: Figure S5), with extensive use of published information from genetic or biochemical studies regarding observed mutant phenotypes or biochemical characteristics of the proteins involved.

The occurrence and expression changes of ERFs, PIL5, and GA2 oxidase antisense transcripts were validated by qPCR (Additional file 5: Figure S3). The occurrence of these antisense transcripts during DT can reflect the well-documented antagonism of ABA with ethylene [83]. The presence of antisense transcripts corresponding to PIL5 and the GA degrading enzyme GA2 oxidase that is activated by PIL5 [84] is not readily explicable as an expected repressive effect on GA metabolism during dormancy (Fig. 10). There are conflicting reports on whether PIL5 acts to trigger release from dormancy or inhibit germination [81, 84]. It is possible that this is an instance of a positive regulatory effect of an antisense transcript on RNA stability [49] or that these antisense transcripts are stored for germination to suppress PIL5 and GA2 oxidase to release dormancy.

#### Inferring transcript and protein functions in the context of regulation of seed filling

In Arabidopsis, seed maturation starts with the expression of LEC1, which induces transcription of L1L, LEC2, FUS3, and ABI3 [1, 85]. LEC1 and L1L represent HAP3-type subunits of heterotrimeric CCAAT-box binding factors [86–88], which activate transcription of genes involved in the synthesis and accumulation of seed storage compounds [89–91]. ABI3 and FUS3 are positive regulators of the ABI5 family of TFs, including bZIP66, promoting accumulation of seed storage compounds [1, 92]. L1L, HAP2A, and bZIP66 are components of the B3 network that were alternatively spliced (Additional file 5: Figure S5B). The novel soybean L1L variant is a noncoding antisense transcript confirmed by qPCR to be expressed in the early maturation phase (Additional file 5: Figure S3). Because the L1L transcripts showed negatively correlated expression profiles, it is possible that the antisense transcript negatively regulates levels of the sense transcript in soybean. LEC1-mediated transient activation of L1L [91] could be inhibited post-transcriptionally through this antisense transcript to confine the L1L presence to the early phases of seed filling. Although the L1L and HAP2A proteins physically interact [88, 93, 94], their transcripts were present at different times during soybean embryo development, which makes concurrent protein-protein interactions and transcriptional regulation unlikely. Genetic evidence in Arabidopsis supports the co-regulation of HAP2A and LEC1/ L1L transcription in early seed filling [95]. These two HAP2A isoforms are probably not the bona-fide L1L interacting partners in soybean.

The B3 network contains transcriptional regulators. Activities of these TFs are also regulated at the post-transcriptional level. Several ABA-related and/or FUS3-regulated genes encoding protein kinases (BAM2, CPK11) and components of the 26S proteasome (DRIP2 and RAD23) were alternatively spliced and, in some cases, could be associated with specific TFs (Additional file 12: Figure S5B). Arabidopsis Ca^2+^-dependent protein kinase CPK11 acts in parallel with SnRK2 kinases to phosphorylate and activate specific bZIP TFs (ABFs, ABI3, and ABI5) involved in promoting dormancy [68, 75, 96, 97]. The full-length CPK11 (Glyma06g16920.1) strongly co-expressed with L1L and several FUS3-regulated metabolic genes. Both BAM2 transcripts, SCPL17, and AP2/B3 TF were also identified as the nearest neighbors of CPK11 regulated by FUS3. While SCPL17 and AP2/B3 are functionally uncharacterized, BAM2 is involved in flower and fruit development [98, 99]. Association of these transcripts with CPK11 and FUS3 implicates their potential involvement in early seed filling signaling.

Interestingly, the novel CPK11 SV was expressed during mid-to-late seed filling and lacked both EFh domains present in the canonical SV. The EFh domains occur as pairs and are responsible for changing protein conformation upon Ca^2+^ binding to modulate protein activity [100]. The novel CPK11 variant could phosphorylate its targets during mid-to-late seed filling independently of Ca^2+^-mediated signaling. Its direct co-expressers AFP2 and DRIP2 are associated with the B3 network through ABA/ABI3/ABI5 and FUS3 signaling, respectively. DRIP2 ubiquitinates the positive regulator of ABA-independent drought responses DREB2A [101–103]. Protein-protein interactions of the novel DRIP2 SV are likely compromised due to the absence of the C3HC4-type RING-finger domain and functions of this SV in the B3 network and early seed filling phases remain to be elucidated.

RAD23 proteins are similar to UBQ and are involved in transporting ubiquitinated proteins to the 26S proteasome for degradation [104, 105]. RAD23 transcription was suppressed in response to ABA and in the protein phosphatase 2C *abi1* mutant [106]. In addition, RAD23 was identified as an interacting partner of a rice ABI3 homolog [107], placing it downstream of FUS3, SnRKs, and CPK11 in the B3 network as a negative regulator of ABI3 activity. The known RAD23B SV belonged to the early super-cluster and could be involved in delivering ABI3 for degradation to the 26S proteasome in developing soybean embryos. It is not clear whether the novel SV was active as it lacked a UBQ superfamily domain [108].

## Conclusions

This report demonstrates the usefulness of our integrated computational framework for the analysis of transcriptomics data, leading to prediction of experimentally testable and specific hypotheses concerning the functions of expressed transcripts. The behavior of many of the coding transcripts identified here has not been studied previously in seeds or at all in the case of long noncoding and antisense RNAs. Taken together, a common functional theme integrates the hubs related to DT in regulation, stress responses, and phytohormone signaling and suggests the existence of distinct ABA-related pathways, specific to different phases of soybean seed development. Several components of the B3 regulatory seed filling network were subjected to AS, potentially leading to differential expression and regulation as well as novel functionalities. Our computational approaches facilitated identification of other regulators possibly involved in seed filling and desiccation and dormancy induction phases of soybean embryo development.

## Availability of supporting data

The raw data and differential expression results are available at GEO (GSE46153). All other high-throughput data are available in Additional files.

## Additional files

Additional file 1: Figure S1. RNA-Seq data and other computational analysis pipelines. The tools are shown in red, classifier specific tools are in green, transcript classes are in yellow, and outputs are in white. (PPTX 101 kb) Additional file 2:
**“BatchTranslator.py” program.** (PY 6 kb) Additional file 3: Figure S2. Changes in transcript levels in developing soybean embryos. The set of 2,938 transcripts were clustered using k-means algorithm into 25 clusters based on the changes in their expression within 10 developmental time points (5, 10, 15, 20, 15, 30, 35, 40, 45, and 55 days after marking and each time point corresponds to each column; rows are individual transcripts). The numbers in the parentheses indicate the number of transcripts present in each cluster. Red color indicates high expression and green color low or no expression. (PPTX 277 kb) Additional file 4: Table S1. Sense and antisense transcripts and primers chosen for validation of RNA-Seq-based expression level changes. Sense and antisense transcripts are shown with the corresponding annotation, primer pairs used for qPCR, time points of differential expression, and notes on the presence of additional melt curve peaks. (PPTX 39 kb) Additional file 5: Figure S3. Quantitative (qPCR) results for selected sense and antisense transcript pairs. qPCR was performed using specific primers (Additional file 4: Table S1) for each transcript as described [25]. With the exception of L1L sense, which showed expression at day 45 based on qPCR, but not RNA-Seq results, all tested transcripts showed a good agreement in transcript changes between these two methods. L1L is known to be expressed only during early seed filling stages [85] and absent in desiccating embryos, suggesting that the qPCR signal at day 45 came from an unknown template. In the case of PIL5, only antisense transcript could be validated, as there were several known and novel sense PIL5 transcripts detected by RNA-Seq with no unique sequences to distinguish them by qPCR. (PPTX 71 kb) Additional file 6: Table S2. Domains in all expressed transcripts. Batch CD-Search was used for large-scale conserved domain search of all expressed transcripts during soybean embryo development. (XLSX 22764 kb) Additional file 7:
**The “noncoding.fa” file.** This FASTA file contains all lncRNA transcripts and their sequences identified in developing soybean embryos. (FA 32275 kb) Additional file 8: Table S3. Summary of all information relevant to 2,938 transcripts. This file contains the following information: gene and transcript IDs, k-means and super-cluster assignments, various transcript features related to sequence length, conserved domains, CPC and CodeWise results related to coding potential, and Mercator-based annotation. Most information in other supplemental Tables is derived from this file to facilitate further data mining. (XLSX 1589 kb) Additional file 9: Figure S4. Distribution of 2,938 transcripts among the super-clusters. The graph shows the distribution of known and novel transcripts belonging to different Cuffcompare classes among six super-clusters. (PPTX 367 kb) Additional file 10: Table S4. Pearson correlations between sense and antisense transcript pairs. (A) All identified sense and antisense transcript pairs. (B) Correlations between known sense (class “=”) and antisense (classes “x” and “s”) transcripts. (XLSX 199 kb) Additional file 11: Table S5. Transcripts belonging to ABA-related genes and FUS3 regulon. Arabidopsis genes were obtained from [1, 51]. Mercator output for soybean transcripts was parsed to obtain potential Arabidopsis homologs. (A) These two datasets were cross-referenced to identify transcripts that were related to at least one of these regulators. (B) ABA-related transcripts; color-coding corresponds to standard assigned super-cluster colors. (XLSX 271 kb) Additional file 12: Figure S5. The participation of transcripts in proposed signaling pathways involved in ABA- and FUS3-related responses. (A) ABA responsive SVs unique to DT with associated functions. NCED5 probably synthesizes ABA during DT. RCAR3 is a well-studied component of ABA receptor complex leading to signaling mediated by several ABA-related components subjected to AS, although their relationships and interactions are not clear (red arrows). GCR1 is an ABA responsive G protein-coupled receptor that was connected to 38 transcripts, some of which are already known to be associated with ABA signaling leading to dormancy (Fig. 9a and b). Possible interactions between the two receptors are not established as yet. (B) FUS3 regulon of transcripts related to the inferred B3 network in developing soybean embryos. These signaling pathways were drawn in the Beacon editor. Signaling components subjected to AS are shown in black boxes accompanying the relevant activities. (PPTX 303 kb) Additional file 13: Table S6. GCR1 and CPK11 sub-networks. The nearest neighbors of the known and novel GCR1 and CPK11 SVs were visualized in Cytoscape from the ArrayMining network. The following sub-networks are presented: (A) Known GCR1 SV. (B) Novel GCR1 SV. (C) Known CPK11 SV. (D) Novel CPK11 SV. (XLSX 77 kb)

## Abbreviations

ABA: Abscisic acid
ABI: ABSCISIC ACID INSENSITIVE
AREB: ABA-responsive element binding
AFP: ABI5-binding protein
AP: APETALA
ARF: Auxin response factor
AS: Alternative splicing
AUC: Area under the receiver operating characteristic curve
BAM: Barely any meristem
BLAST: Basic Local Alignment Search Tool
CBS: Cystathionine beta-synthase
CDD: Conserved domain database
CPAT: Coding Potential Assessment Tool
CPC: Coding Potential Calculator
CPK: Calcium-dependent protein kinase
DRIP: DREB2A-interacting protein
DT: Desiccation tolerance
EFh: EF-hand
ERF: Ethylene response factor
FDR: False discovery rate
FPKM: Fragments per kilobase of exon per million fragments mapped
FUS: FUSCA
GA: Gibberellin
GAPDH: Glyceraldehyde-3-phosphate dehydrogenase
GCR: G-PROTEIN COUPLED RECEPTOR
GDP: Guanosine diphosphate
GME: GDP-mannose-3,5-epimerase
GUN: Genome-uncoupled
HDAC: Histone deacetylase
Hsp: Heat shock protein
KOG: Eukaryotic Orthologous Groups
L1L: LEC1-LIKE
LEC: LEAFY COTYLEDON
lincRNA: Long intergenic noncoding RNA
lncRNA: Long noncoding RNA
NADPH: Reduced nicotinamide adenine dinucleotide phosphate
NCED: 9-cis-epoxycarotenoid dioxygenase
ncRNA: Noncoding RNA
NF: Nuclear factor
NAC: NAM domain-containing
NAM: No apical meristem
ORF: Open reading frame
PC: Principal component
PCA: Principal component analysis
PIL: Phytochrome-interacting basic helix-loop-helix
PLD: Phospholipase D
pre-mRNA: Premature messenger RNA
PYL: PYRABACTIN RESISTANCE 1-LIKE
qPCR: Quantitative Polymerase Chain Reaction
RAD: Radiation sensitive
RBF: Radial basis functional
RCAR: REGULATORY COMPONENTS OF ABA RECEPTOR
RING: Really Interesting Gene
RNA-Seq: RNA sequencing
SAM MT: S-adenosyl-L-methionine methyltransferase
SCPL: Ser carboxypeptidase-like
SnRK: sucrose nonfermenting-related kinase
SPCH: SPEECHLESS
SV: Splice variant
SVM: Support vector machine
TAIR: The Arabidopsis Information Resource
TF: Transcription factor
THIC: Thiamine biosynthesis protein C
UBQ: Ubiquitin
UTR: Untranslated region

## References

[1] Finkelstein R (2013). Abscisic acid synthesis and response. Arabidopsis Book.

[2] Santos-Mendoza M, Dubreucq B, Baud S, Parcy F, Caboche M, Lepiniec L (2008). Deciphering gene regulatory networks that control seed development and maturation in Arabidopsis. Plant J.

[3] Angelovici R, Galili G, Fernie AR, Fait A (2010). Seed desiccation: a bridge between maturation and germination. Trends Plant Sci.

[4] Finkelstein R, Reeves W, Ariizumi T, Steber C (2008). Molecular aspects of seed dormancy. Annu Rev Plant Biol.

[5] Gutierrez L, Van Wuytswinkel O, Castelain M, Bellini C (2007). Combined networks regulating seed maturation. Trends Plant Sci.

[6] Ponjavic J, Ponting CP, Lunter G (2007). Functionality or transcriptional noise? Evidence for selection within long noncoding RNAs. Genome Res.

[7] Yang L, Froberg JE, Lee JT (2014). Long noncoding RNAs: fresh perspectives into the RNA world. Trends Biochem Sci.

[8] Andrews SJ, Rothnagel JA (2014). Emerging evidence for functional peptides encoded by short open reading frames. Nat Rev Genet.

[9] Lindsey K, Casson S, Chilley P (2002). Peptides: new signalling molecules in plants. Trends Plant Sci.

[10] Guttman M, Russell P, Ingolia NT, Weissman JS, Lander ES (2013). Ribosome profiling provides evidence that large noncoding RNAs do not encode proteins. Cell.

[11] Wilusz JE, Sunwoo H, Spector DL (2009). Long noncoding RNAs: functional surprises from the RNA world. Genes Dev.

[12] Amor BB, Wirth S, Merchan F, Laporte P, d’Aubenton-Carafa Y, Hirsch J (2009). Novel long non-protein coding RNAs involved in Arabidopsis differentiation and stress responses. Genome Res.

[13] Zhang W, Han Z, Guo Q, Liu Y, Zheng Y, Wu F (2014). Identification of maize long non-coding RNAs responsive to drought stress. PLoS One.

[14] Boerner S, McGinnis KM (2012). Computational identification and functional predictions of long noncoding RNA in *Zea mays*. PLoS One.

[15] Bardou F, Ariel F, Simpson CG, Romero-Barrios N, Laporte P, Balzergue S (2014). Long noncoding RNA modulates alternative splicing regulators in Arabidopsis. Dev Cell.

[16] Apweiler R, Attwood TK, Bairoch A, Bateman A, Birney E, Biswas M (2001). The InterPro database, an integrated documentation resource for protein families, domains and functional sites. Nucleic Acids Res.

[17] Marchler-Bauer A, Lu S, Anderson JB, Chitsaz F, Derbyshire MK, DeWeese-Scott C (2011). CDD: a Conserved Domain Database for the functional annotation of proteins. Nucleic Acids Res.

[18] Kong L, Zhang Y, Ye Z-Q, Liu X-Q, Zhao S-Q, Wei L (2007). CPC: assess the protein-coding potential of transcripts using sequence features and support vector machine. Nucleic Acids Res.

[19] Jin J, Liu J, Wang H, Wong L, Chua N-H (2013). PLncDB: plant long non-coding RNA database. Bioinformatics.

[20] Lin MF, Jungreis I, Kellis M (2011). PhyloCSF: a comparative genomics method to distinguish protein coding and non-coding regions. Bioinformatics.

[21] Sun K, Chen X, Jiang P, Song X, Wang H, Sun H (2013). iSeeRNA: identification of long intergenic non-coding RNA transcripts from transcriptome sequencing data. BMC Genomics.

[22] Chew G-L, Pauli A, Rinn JL, Regev A, Schier AF, Valen E (2013). Ribosome profiling reveals resemblance between long non-coding RNAs and 5′ leaders of coding RNAs. Development.

[23] Lu ZJ, Yip KY, Wang G, Shou C, Hillier LW, Khurana E (2011). Prediction and characterization of noncoding RNAs in *C. elegans* by integrating conservation, secondary structure, and high-throughput sequencing and array data. Genome Res.

[24] Torarinsson E, Sawera M, Havgaard JH, Fredholm M, Gorodkin J (2006). Thousands of corresponding human and mouse genomic regions unalignable in primary sequence contain common RNA structure. Genome Res.

[25] Aghamirzaie D, Nabiyouni M, Fang Y, Klumas C, Heath LS, Grene R (2013). Changes in RNA splicing in developing soybean (*Glycine max*) embryos. Biology.

[26] Collakova E, Aghamirzaie D, Fang Y, Klumas C, Tabataba F, Kakumanu A (2013). Metabolic and transcriptional reprogramming in developing soybean (*Glycine max*) embryos. Metabolites.

[27] Trapnell C, Roberts A, Goff L, Pertea G, Kim D, Kelley DR (2012). Differential gene and transcript expression analysis of RNA-seq experiments with TopHat and Cufflinks. Nat Protoc.

[28] Trapnell C, Williams BA, Pertea G, Mortazavi A, Kwan G, van Baren MJ (2010). Transcript assembly and quantification by RNA-Seq reveals unannotated transcripts and isoform switching during cell differentiation. Nat Biotechnol.

[29] Lohse M, Nagel A, Herter T, May P, Schroda M, Zrenner R (2014). Mercator: a fast and simple web server for genome scale functional annotation of plant sequence data. Plant Cell Environ.

[30] Hofacker IL (2003). Vienna RNA, secondary structure server. Nucleic Acids Res.

[31] UniProt C (2015). UniProt: a hub for protein information. Nucleic Acids Res.

[32] Lamesch P, Berardini TZ, Li D, Swarbreck D, Wilks C, Sasidharan R (2012). The Arabidopsis Information Resource (TAIR): improved gene annotation and new tools. Nucleic Acids Res.

[33] Goodstein DM, Shu S, Howson R, Neupane R, Hayes RD, Fazo J (2012). Phytozome: a comparative platform for green plant genomics. Nucleic Acids Res.

[34] Chang C-C, Lin C-J (2011). LIBSVM: a library for support vector machines. ACM Trans Intell Syst Technol.

[35] Xie C, Yuan J, Li H, Li M, Zhao G, Bu D (2014). NONCODEv4: exploring the world of long non-coding RNA genes. Nucleic Acids Res.

[36] de Hoon MJL, Imoto S, Nolan J, Miyano S (2004). Open source clustering software. Bioinformatics.

[37] Saldanha AJ (2004). Java Treeview - extensible visualization of microarray data. Bioinformatics.

[38] Glaab E, Garibaldi JM, Krasnogor N (2009). ArrayMining: a modular web-application for microarray analysis combining ensemble and consensus methods with cross-study normalization. BMC Bioinf.

[39] Shannon P, Markiel A, Ozier O, Baliga NS, Wang JT, Ramage D (2003). Cytoscape: a software environment for integrated models of biomolecular interaction networks. Genome Res.

[40] Kakumanu A, Ambavaram MM, Klumas C, Krishnan A, Batlang U, Myers E (2012). Effects of drought on gene expression in maize reproductive and leaf meristem tissue revealed by RNA-Seq. Plant Physiol.

[41] Le Novere N, Hucka M, Mi H, Moodie S, Schreiber F, Sorokin A (2009). The systems biology graphical notation. Nat Biotechnol.

[42] Zhang X, Lu X, Shi Q, Xu XQ, Leung HC, Harris LN (2006). Recursive SVM feature selection and sample classification for mass-spectrometry and microarray data. BMC Bioinformatics.

[43] Bhasin M, Raghava GP (2004). ESLpred: SVM-based method for subcellular localization of eukaryotic proteins using dipeptide composition and PSI-BLAST. Nucleic Acids Res.

[44] Crawford BC, Yanofsky MF (2011). HALF FILLED promotes reproductive tract development and fertilization efficiency in *Arabidopsis thaliana*. Development.

[45] Bánfai B, Jia H, Khatun J, Wood E, Risk B, Gundling WE (2012). Long noncoding RNAs are rarely translated in two human cell lines. Genome Res.

[46] Thimm O, Bläsing O, Gibon Y, Nagel A, Meyer S, Krüger P (2004). Mapman: a user‐driven tool to display genomics data sets onto diagrams of metabolic pathways and other biological processes. Plant J.

[47] Derrien T, Johnson R, Bussotti G, Tanzer A, Djebali S, Tilgner H (2012). The GENCODE v7 catalog of human long noncoding RNAs: analysis of their gene structure, evolution, and expression. Genome Res.

[48] Kung JT, Colognori D, Lee JT (2013). Long noncoding RNAs: past, present, and future. Genetics.

[49] Jabnoune M, Secco D, Lecampion C, Robaglia C, Shu Q, Poirier Y (2013). A rice cis-natural antisense RNA acts as a translational enhancer for its cognate mRNA and contributes to phosphate homeostasis and plant fitness. Plant Cell.

[50] Wang H, Chung PJ, Liu J, Jang IC, Kean MJ, Xu J (2014). Genome-wide identification of long noncoding natural antisense transcripts and their responses to light in Arabidopsis. Genome Res.

[51] Wang F, Perry SE (2013). Identification of direct targets of FUSCA3, a key regulator of Arabidopsis seed development. Plant Physiol.

[52] Pillitteri LJ, Bemis SM, Shpak ED, Torii KU (2007). Haploinsufficiency after successive loss of signaling reveals a role for ERECTA-family genes in Arabidopsis ovule development. Development.

[53] Wang L, Hua D, He J, Duan Y, Chen Z, Hong X (2011). Auxin Response Factor2 (ARF2) and its regulated homeodomain gene HB33 mediate abscisic acid response in Arabidopsis. PLoS Genet.

[54] Wu MF, Tian Q, Reed JW (2006). Arabidopsis microRNA167 controls patterns of ARF6 and ARF8 expression, and regulates both female and male reproduction. Development.

[55] Diaz-Meco MT, Moscat J (2001). MEK5, a new target of the atypical protein kinase C isoforms in mitogenic signaling. Mol Cell Biol.

[56] Ito T, Matsui Y, Ago T, Ota K, Sumimoto H (2001). Novel modular domain PB1 recognizes PC motif to mediate functional protein-protein interactions. EMBO J.

[57] Terasawa H, Noda Y, Ito T, Hatanaka H, Ichikawa S, Ogura K (2001). Structure and ligand recognition of the PB1 domain: a novel protein module binding to the PC motif. EMBO J.

[58] Metzger MB, Hristova VA, Weissman AM (2012). HECT and RING finger families of E3 ubiquitin ligases at a glance. J Cell Sci.

[59] Du SY, Zhang XF, Lu Z, Xin Q, Wu Z, Jiang T (2012). Roles of the different components of magnesium chelatase in abscisic acid signal transduction. Plant Mol Biol.

[60] Mochizuki N, Brusslan JA, Larkin R, Nagatani A, Chory J (2001). Arabidopsis genomes uncoupled 5 (GUN5) mutant reveals the involvement of Mg-chelatase H subunit in plastid-to-nucleus signal transduction. Proc Natl Acad Sci U S A.

[61] Wang YX, Huang H (2014). Review on statistical methods for gene network reconstruction using expression data. J Theor Biol.

[62] Zhang S, Jin G, Zhang XS, Chen L (2007). Discovering functions and revealing mechanisms at molecular level from biological networks. Proteomics.

[63] Hadden DA, Phillipson BA, Johnston KA, Brown LA, Manfield IW, El-Shami M (2006). Arabidopsis PEX19 is a dimeric protein that binds the peroxin PEX10. Mol Membr Biol.

[64] Labusch C, Shishova M, Effendi Y, Li M, Wang X, Scherer GF (2013). Patterns and timing in expression of early auxin-induced genes imply involvement of phospholipases A (pPLAs) in the regulation of auxin responses. Mol Plant.

[65] Kuroda H, Yanagawa Y, Takahashi N, Horii Y, Matsui M (2012). A comprehensive analysis of interaction and localization of Arabidopsis SKP1-like (ASK) and F-box (FBX) proteins. PLoS One.

[66] Umezawa T, Sugiyama N, Takahashi F, Anderson JC, Ishihama Y, Peck SC (2013). Genetics and phosphoproteomics reveal a protein phosphorylation network in the abscisic acid signaling pathway in *Arabidopsis thaliana*. Sci Signal.

[67] Warpeha KM, Upadhyay S, Yeh J, Adamiak J, Hawkins SI, Lapik YR (2007). The GCR1, GPA1, PRN1, NF-Y signal chain mediates both blue light and abscisic acid responses in Arabidopsis. Plant Physiol.

[68] Zhu SY, Yu XC, Wang XJ, Zhao R, Li Y, Fan RC (2007). Two calcium-dependent protein kinases, CPK4 and CPK11, regulate abscisic acid signal transduction in Arabidopsis. Plant Cell.

[69] Fang L, Hou X, Lee LY, Liu L, Yan X, Yu H (2011). AtPV42a and AtPV42b redundantly regulate reproductive development in *Arabidopsis thaliana*. PLoS One.

[70] Gissot L, Polge C, Jossier M, Girin T, Bouly JP, Kreis M (2006). AKINbetagamma contributes to SnRK1 heterotrimeric complexes and interacts with two proteins implicated in plant pathogen resistance through its KIS/GBD sequence. Plant Physiol.

[71] Di C, Yuan J, Wu Y, Li J, Lin H, Hu L (2014). Characterization of stress-responsive lncRNAs in *Arabidopsis thaliana* by integrating expression, epigenetic and structural features. Plant J.

[72] Guillen G, Diaz-Camino C, Loyola-Torres CA, Aparicio-Fabre R, Hernandez-Lopez A, Diaz-Sanchez M (2013). Detailed analysis of putative genes encoding small proteins in legume genomes. Front Plant Sci.

[73] Jorgensen RA, Dorantes-Acosta AE (2012). Conserved peptide upstream open reading frames are associated with regulatory genes in angiosperms. Front Plant Sci.

[74] James AB, Syed NH, Bordage S, Marshall J, Nimmo GA, Jenkins GI (2012). Alternative splicing mediates responses of the Arabidopsis circadian clock to temperature changes. Plant Cell.

[75] Lynch T, Erickson BJ, Finkelstein RR (2012). Direct interactions of ABA-insensitive(ABI)-clade protein phosphatase(PP)2Cs with calcium-dependent protein kinases and ABA response element-binding bZIPs may contribute to turning off ABA response. Plant Mol Biol.

[76] Tsai AY, Gazzarrini S (2012). AKIN10 and FUSCA3 interact to control lateral organ development and phase transitions in Arabidopsis. Plant J.

[77] Lu QS, Paz JD, Pathmanathan A, Chiu RS, Tsai AY, Gazzarrini S (2010). The C-terminal domain of FUSCA3 negatively regulates mRNA and protein levels, and mediates sensitivity to the hormones abscisic acid and gibberellic acid in Arabidopsis. Plant J.

[78] Stone SL (2014). The role of ubiquitin and the 26S proteasome in plant abiotic stress signaling. Front Plant Sci.

[79] Bhaskara GB, Nguyen TT, Verslues PE (2012). Unique drought resistance functions of the highly ABA-induced clade A protein phosphatase 2Cs. Plant Physiol.

[80] Schweighofer A, Hirt H, Meskiene I (2004). Plant PP2C phosphatases: emerging functions in stress signaling. Trends Plant Sci.

[81] Graeber K, Nakabayashi K, Miatton E, Leubner-Metzger G, Soppe WJ (2012). Molecular mechanisms of seed dormancy. Plant Cell Environ.

[82] Saavedra X, Modrego A, Rodriguez D, Gonzalez-Garcia MP, Sanz L, Nicolas G (2010). The nuclear interactor PYL8/RCAR3 of *Fagus sylvatica* FsPP2C1 is a positive regulator of abscisic acid signaling in seeds and stress. Plant Physiol.

[83] Holdsworth MJ, Bentsink L, Soppe WJ (2008). Molecular networks regulating Arabidopsis seed maturation, after-ripening, dormancy and germination. New Phytol.

[84] Oh E, Yamaguchi S, Kamiya Y, Bae G, Chung WI, Choi G (2006). Light activates the degradation of PIL5 protein to promote seed germination through gibberellin in Arabidopsis. Plant J.

[85] Kwong RW, Bui AQ, Lee H, Kwong LW, Fischer RL, Goldberg RB (2003). LEAFY COTYLEDON1-LIKE defines a class of regulators essential for embryo development. Plant Cell.

[86] Calvenzani V, Testoni B, Gusmaroli G, Lorenzo M, Gnesutta N, Petroni K (2012). Interactions and CCAAT-binding of *Arabidopsis thaliana* NF-Y subunits. PLoS One.

[87] Lee H, Fischer RL, Goldberg RB, Harada JJ (2003). Arabidopsis LEAFY COTYLEDON1 represents a functionally specialized subunit of the CCAAT binding transcription factor. Proc Natl Acad Sci U S A.

[88] Yazawa K, Kamada H (2007). Identification and characterization of carrot HAP factors that form a complex with the embryo-specific transcription factor C-LEC1. J Exp Bot.

[89] Braybrook SA, Harada JJ (2008). LECs go crazy in embryo development. Trends Plant Sci.

[90] Kagaya Y, Okuda R, Ban A, Toyoshima R, Tsutsumida K, Usui H (2005). Indirect ABA-dependent regulation of seed storage protein genes by FUSCA3 transcription factor in Arabidopsis. Plant Cell Physiol.

[91] Mendes A, Kelly AA, van Erp H, Shaw E, Powers SJ, Kurup S (2013). bZIP67 regulates the omega-3 fatty acid content of Arabidopsis seed oil by activating fatty acid desaturase3. Plant Cell.

[92] Bensmihen S, Giraudat J, Parcy F (2005). Characterization of three homologous basic leucine zipper transcription factors (bZIP) of the ABI5 family during *Arabidopsis thaliana* embryo maturation. J Exp Bot.

[93] Mostafavi S, Ray D, Warde-Farley D, Grouios C, Morris Q (2008). GeneMANIA: a real-time multiple association network integration algorithm for predicting gene function. Genome Biol.

[94] Zuberi K, Franz M, Rodriguez H, Montojo J, Lopes CT, Bader GD (2013). GeneMANIA prediction server 2013 update. Nucleic Acids Res.

[95] Mu J, Tan H, Hong S, Liang Y, Zuo J (2013). Arabidopsis transcription factor genes NF-YA1, 5, 6, and 9 play redundant roles in male gametogenesis, embryogenesis, and seed development. Mol Plant.

[96] Uno Y, Furihata T, Abe H, Yoshida R, Shinozaki K, Yamaguchi-Shinozaki K (2000). Arabidopsis basic leucine zipper transcription factors involved in an abscisic acid-dependent signal transduction pathway under drought and high-salinity conditions. Proc Natl Acad Sci U S A.

[97] Yoshida T, Fujita Y, Maruyama K, Mogami J, Todaka D, Shinozaki K (2015). Four Arabidopsis AREB/ABF transcription factors function predominantly in gene expression downstream of SnRK2 kinases in abscisic acid signalling in response to osmotic stress. Plant Cell Environ.

[98] DeYoung BJ, Bickle KL, Schrage KJ, Muskett P, Patel K, Clark SE (2006). The CLAVATA1-related BAM1, BAM2 and BAM3 receptor kinase-like proteins are required for meristem function in Arabidopsis. Plant J.

[99] Durbak AR, Tax FE (2011). CLAVATA signaling pathway receptors of Arabidopsis regulate cell proliferation in fruit organ formation as well as in meristems. Genetics.

[100] Grabarek Z (2006). Structural basis for diversity of the EF-hand calcium-binding proteins. J Mol Biol.

[101] Liu Q, Kasuga M, Sakuma Y, Abe H, Miura S, Yamaguchi-Shinozaki K (1998). Two transcription factors, DREB1 and DREB2, with an EREBP/AP2 DNA binding domain separate two cellular signal transduction pathways in drought- and low-temperature-responsive gene expression, respectively, in Arabidopsis. Plant Cell.

[102] Mizoi J, Ohori T, Moriwaki T, Kidokoro S, Todaka D, Maruyama K (2013). GmDREB2A;2, a canonical DEHYDRATION-RESPONSIVE ELEMENT-BINDING PROTEIN2-type transcription factor in soybean, is posttranslationally regulated and mediates dehydration-responsive element-dependent gene expression. Plant Physiol.

[103] Qin F, Sakuma Y, Tran LS, Maruyama K, Kidokoro S, Fujita Y (2008). Arabidopsis DREB2A-interacting proteins function as RING E3 ligases and negatively regulate plant drought stress-responsive gene expression. Plant Cell.

[104] Farmer LM, Book AJ, Lee KH, Lin YL, Fu H, Vierstra RD (2010). The RAD23 family provides an essential connection between the 26S proteasome and ubiquitylated proteins in Arabidopsis. Plant Cell.

[105] Fatimababy AS, Lin YL, Usharani R, Radjacommare R, Wang HT, Tsai HL (2010). Cross-species divergence of the major recognition pathways of ubiquitylated substrates for ubiquitin/26S proteasome-mediated proteolysis. FEBS J.

[106] Hoth S, Morgante M, Sanchez JP, Hanafey MK, Tingey SV, Chua NH (2002). Genome-wide gene expression profiling in *Arabidopsis thaliana* reveals new targets of abscisic acid and largely impaired gene regulation in the *abi1*-*1* mutant. J Cell Sci.

[107] Schultz TF, Quatrano RS (1997). Characterization and expression of a rice RAD23 gene. Plant Mol Biol.

[108] Watkins JF, Sung P, Prakash L, Prakash S (1993). The *Saccharomyces cerevisiae* DNA repair gene RAD23 encodes a nuclear protein containing a ubiquitin-like domain required for biological function. Mol Cell Biol.

