# Supplementary material for: Transcriptome-wide functional characterization reveals novel relationships among differentially expressed transcripts in developing soybean embryos
Source: BMC Genomics. 2015 Nov 14;16:928. doi: 10.1186/s12864-015-2108-x (PMC4647491; doi:10.1186/s12864-015-2108-x)
Supplement: Additional file 1: Figure S1. — RNA-Seq data and other computational analysis pipelines. The tools are shown in red, classifier specific tools are in green, transcript classes are in yellow, and outputs are in white. (PPTX 101 kb) [file 12864_2015_2108_MOESM1_ESM.pptx]

## Slide 1
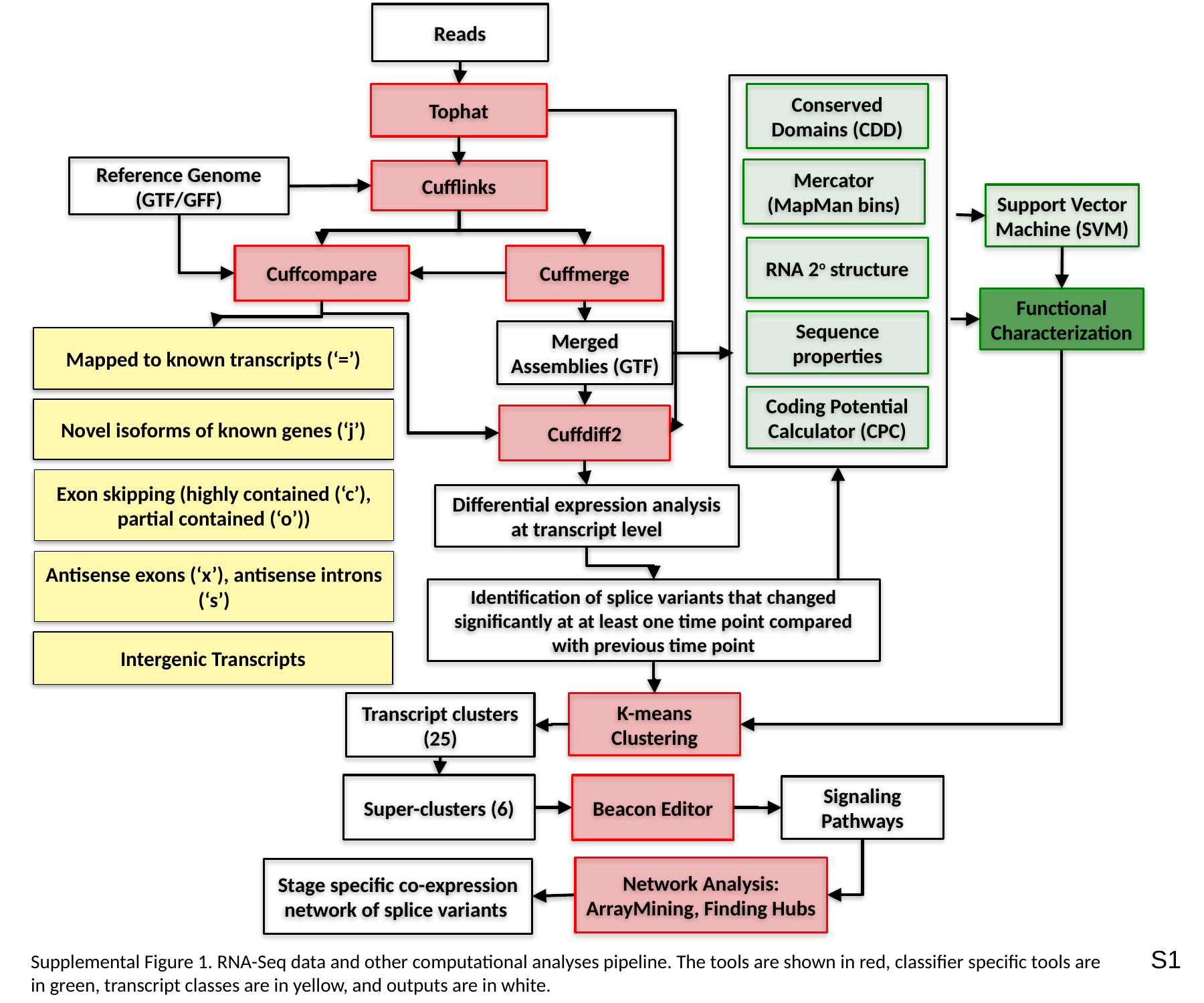

Reads
Tophat
Conserved Domains (CDD)
Mercator (MapMan bins)
RNA 2o structure
Sequence properties
Coding Potential Calculator (CPC)
Reference Genome (GTF/GFF)
Cufflinks
Support Vector Machine (SVM)
Cuffcompare
Cuffmerge
Functional Characterization
Merged Assemblies (GTF)
Mapped to known transcripts (‘=’)
Novel isoforms of known genes (‘j’)
Exon skipping (highly contained (‘c’), partial contained (‘o’))
Antisense exons (‘x’), antisense introns (‘s’)
Intergenic Transcripts
Cuffdiff2
Differential expression analysis at transcript level
Identification of splice variants that changed significantly at at least one time point compared with previous time point
K-means Clustering
Transcript clusters (25)
Beacon Editor
Super-clusters (6)
Signaling Pathways
Network Analysis: ArrayMining, Finding Hubs
Stage specific co-expression network of splice variants
S1
Supplemental Figure 1. RNA-Seq data and other computational analyses pipeline. The tools are shown in red, classifier specific tools are in green, transcript classes are in yellow, and outputs are in white.
