# Supplementary material for: Transcriptome-wide functional characterization reveals novel relationships among differentially expressed transcripts in developing soybean embryos
Source: BMC Genomics. 2015 Nov 14;16:928. doi: 10.1186/s12864-015-2108-x (PMC4647491; doi:10.1186/s12864-015-2108-x)
Supplement: Additional file 3: Figure S2. — Changes in transcript levels in developing soybean embryos. The set of 2,938 transcripts were clustered using k-means algorithm into 25 clusters based on the changes in their expression within 10 developmental time points (5, 10, 15, 20, 15, 30, 35, 40, 45, and 55 days after marking and each time point corresponds to each column; rows are individual transcripts). The numbers in the parentheses indicate the number of transcripts present in each cluster. Red color indicates high expression and green color low or no expression. (PPTX 277 kb) [file 12864_2015_2108_MOESM3_ESM.pptx]

## Slide 1
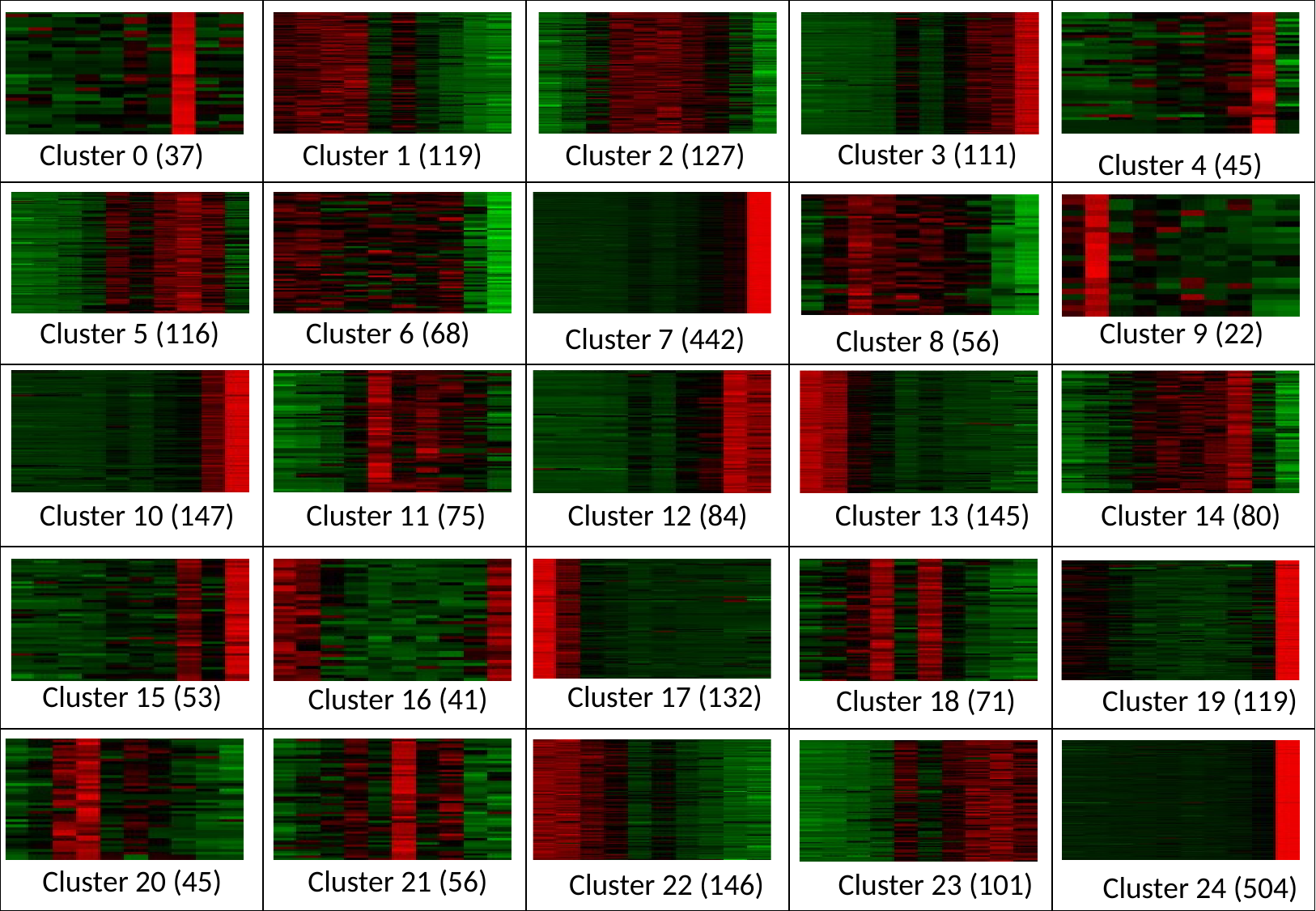

| | | | | |
| --- | --- | --- | --- | --- |
| | | | | |
| | | | | |
| | | | | |
| | | | | |
Cluster 3 (111)
Cluster 0 (37)
Cluster 1 (119)
Cluster 2 (127)
Cluster 4 (45)
Cluster 5 (116)
Cluster 6 (68)
Cluster 9 (22)
Cluster 7 (442)
Cluster 8 (56)
Cluster 11 (75)
Cluster 12 (84)
Cluster 13 (145)
Cluster 14 (80)
Cluster 10 (147)
Cluster 15 (53)
Cluster 17 (132)
Cluster 16 (41)
Cluster 18 (71)
Cluster 19 (119)
Cluster 20 (45)
Cluster 21 (56)
Cluster 22 (146)
Cluster 23 (101)
Cluster 24 (504)

## Slide 2
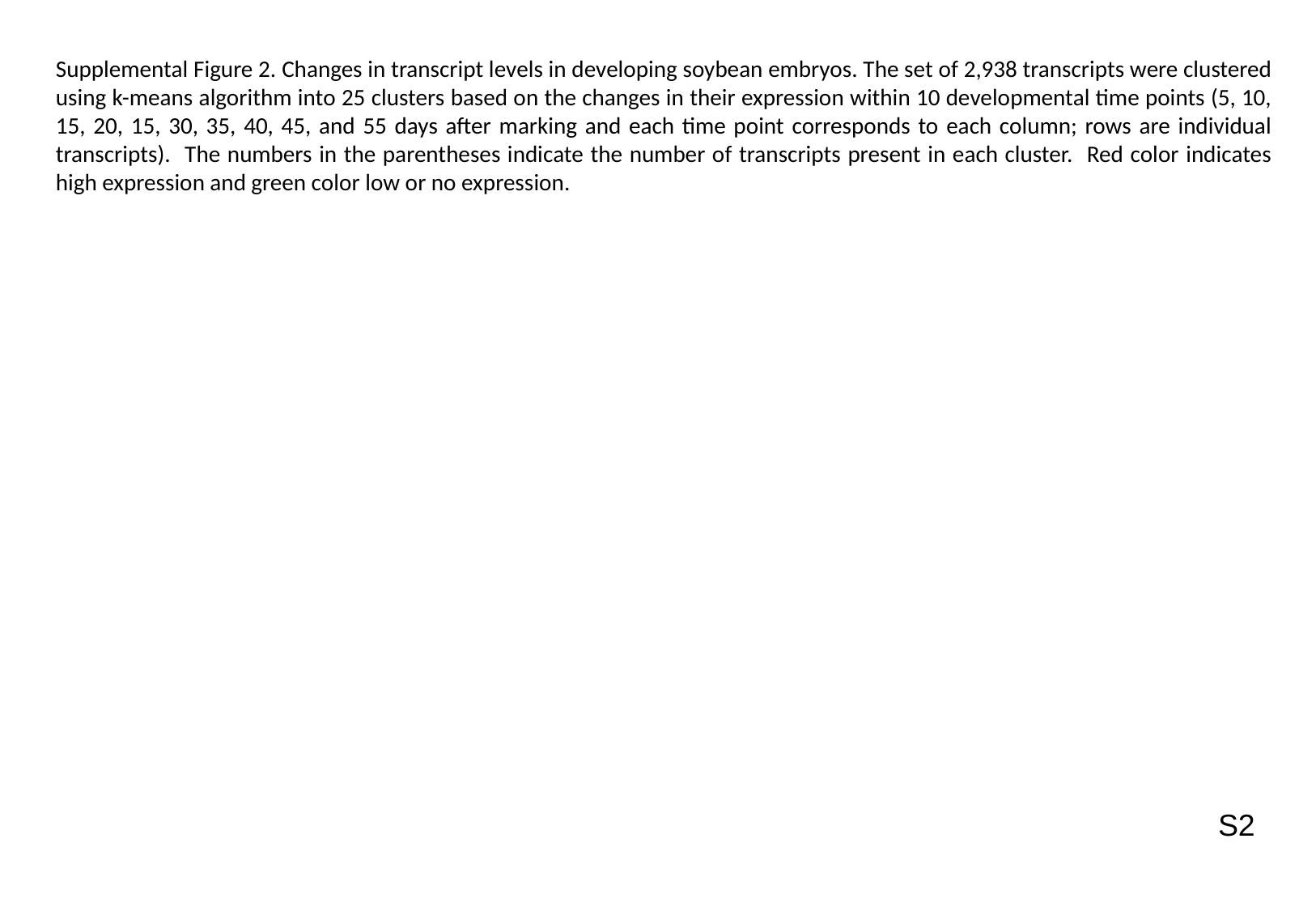

Supplemental Figure 2. Changes in transcript levels in developing soybean embryos. The set of 2,938 transcripts were clustered using k-means algorithm into 25 clusters based on the changes in their expression within 10 developmental time points (5, 10, 15, 20, 15, 30, 35, 40, 45, and 55 days after marking and each time point corresponds to each column; rows are individual transcripts). The numbers in the parentheses indicate the number of transcripts present in each cluster. Red color indicates high expression and green color low or no expression.
S2
