# Supplementary material for: Transcriptome-wide functional characterization reveals novel relationships among differentially expressed transcripts in developing soybean embryos
Source: BMC Genomics. 2015 Nov 14;16:928. doi: 10.1186/s12864-015-2108-x (PMC4647491; doi:10.1186/s12864-015-2108-x)
Supplement: Additional file 4: Table S1. — Sense and antisense transcripts and primers chosen for validation of RNA-Seq-based expression level changes. Sense and antisense transcripts are shown with the corresponding annotation, primer pairs used for qPCR, time points of differential expression, and notes on the presence of additional melt curve peaks. (PPTX 39 kb) [file 12864_2015_2108_MOESM4_ESM.pptx]

## Slide 1
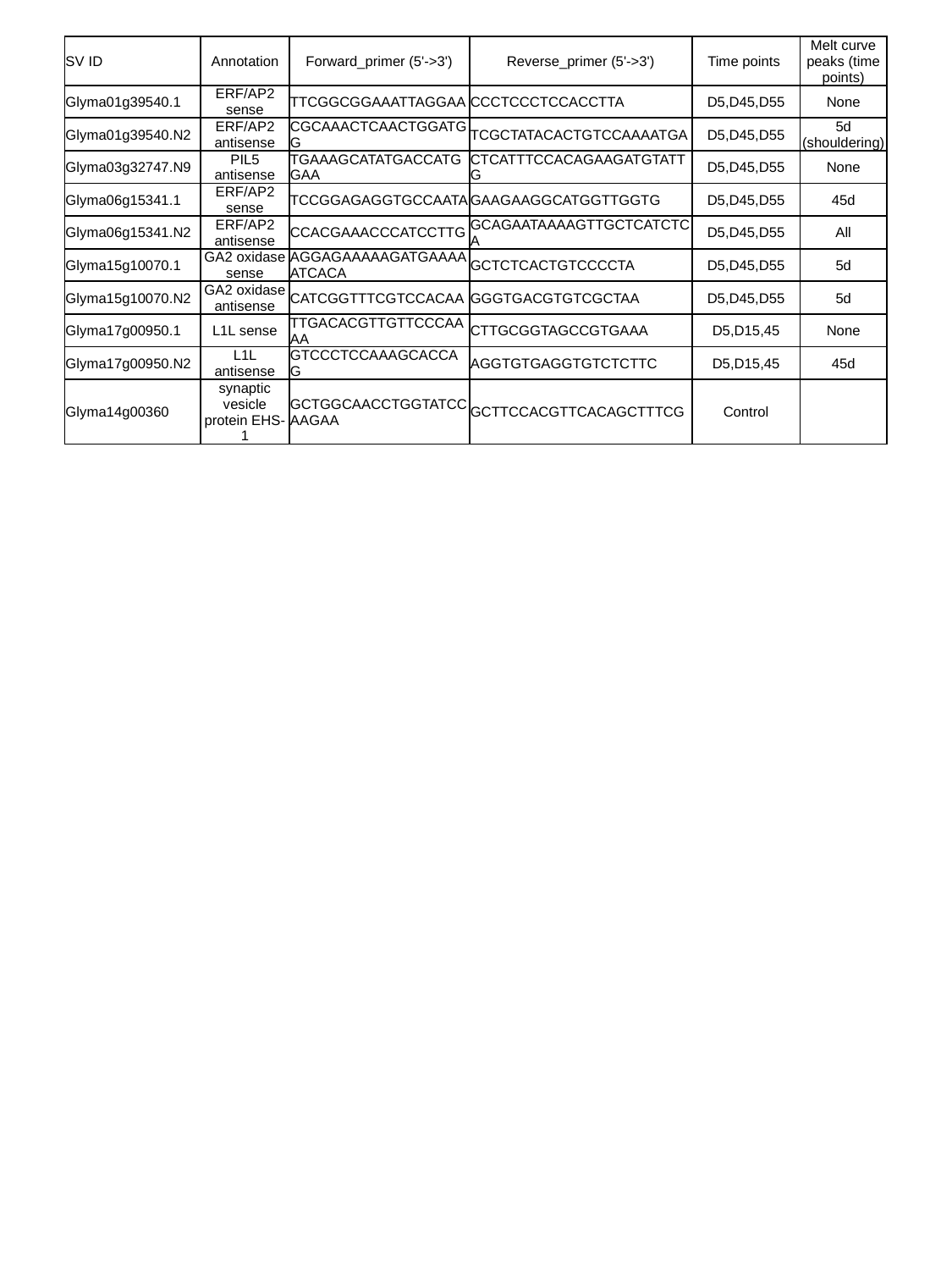

| SV ID | Annotation | Forward\_primer (5'->3') | Reverse\_primer (5'->3') | Time points | Melt curve peaks (time points) |
| --- | --- | --- | --- | --- | --- |
| Glyma01g39540.1 | ERF/AP2 sense | TTCGGCGGAAATTAGGAA | CCCTCCCTCCACCTTA | D5,D45,D55 | None |
| Glyma01g39540.N2 | ERF/AP2 antisense | CGCAAACTCAACTGGATGG | TCGCTATACACTGTCCAAAATGA | D5,D45,D55 | 5d (shouldering) |
| Glyma03g32747.N9 | PIL5 antisense | TGAAAGCATATGACCATGGAA | CTCATTTCCACAGAAGATGTATTG | D5,D45,D55 | None |
| Glyma06g15341.1 | ERF/AP2 sense | TCCGGAGAGGTGCCAATA | GAAGAAGGCATGGTTGGTG | D5,D45,D55 | 45d |
| Glyma06g15341.N2 | ERF/AP2 antisense | CCACGAAACCCATCCTTG | GCAGAATAAAAGTTGCTCATCTCA | D5,D45,D55 | All |
| Glyma15g10070.1 | GA2 oxidase sense | AGGAGAAAAAGATGAAAAATCACA | GCTCTCACTGTCCCCTA | D5,D45,D55 | 5d |
| Glyma15g10070.N2 | GA2 oxidase antisense | CATCGGTTTCGTCCACAA | GGGTGACGTGTCGCTAA | D5,D45,D55 | 5d |
| Glyma17g00950.1 | L1L sense | TTGACACGTTGTTCCCAAAA | CTTGCGGTAGCCGTGAAA | D5,D15,45 | None |
| Glyma17g00950.N2 | L1L antisense | GTCCCTCCAAAGCACCAG | AGGTGTGAGGTGTCTCTTC | D5,D15,45 | 45d |
| Glyma14g00360 | synaptic vesicle protein EHS-1 | GCTGGCAACCTGGTATCCAAGAA | GCTTCCACGTTCACAGCTTTCG | Control | |
