# Supplementary material for: Transcriptome-wide functional characterization reveals novel relationships among differentially expressed transcripts in developing soybean embryos
Source: BMC Genomics. 2015 Nov 14;16:928. doi: 10.1186/s12864-015-2108-x (PMC4647491; doi:10.1186/s12864-015-2108-x)
Supplement: Additional file 5: Figure S3. — Quantitative (qPCR) results for selected sense and antisense transcript pairs. qPCR was performed using specific primers (Additional file 4: Table S1) for each transcript as described [25]. With the exception of L1L sense, which showed expression at day 45 based on qPCR, but not RNA-Seq results, all tested transcripts showed a good agreement in transcript changes between these two methods. L1L is known to be expressed only during early seed filling stages [85] and absent in desiccating embryos, suggesting that the qPCR signal at day 45 came from an unknown template. In the case of PIL5, only antisense transcript could be validated, as there were several known and novel sense PIL5 transcripts detected by RNA-Seq with no unique sequences to distinguish them by qPCR. (PPTX 71 kb) [file 12864_2015_2108_MOESM5_ESM.pptx]

## Slide 1
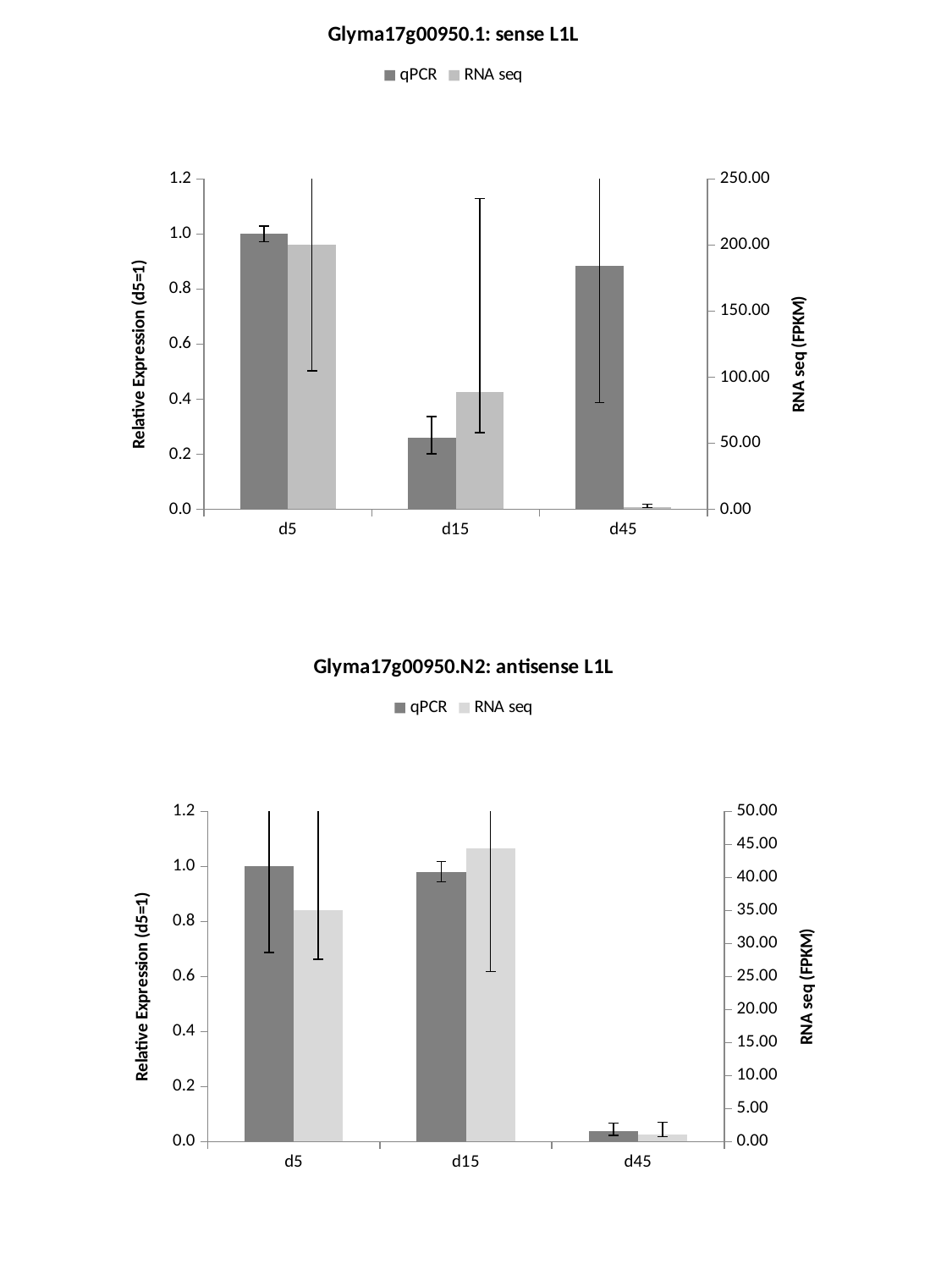

### Chart: Glyma17g00950.1: sense L1L
| Category | | | | |
|---|---|---|---|---|
| d5 | 1.0 | 0.0 | 0.0 | 200.38 |
| d15 | 0.260109424306636 | 0.0 | 0.0 | 88.6077 |
| d45 | 0.883959003259152 | 0.0 | 0.0 | 1.27411 |
### Chart: Glyma17g00950.N2: antisense L1L
| Category | | | | |
|---|---|---|---|---|
| d5 | 1.0 | 0.0 | 0.0 | 35.0056 |
| d15 | 0.98028345519438 | 0.0 | 0.0 | 44.4238 |
| d45 | 0.038502415909791 | 0.0 | 0.0 | 1.1032 |

## Slide 2
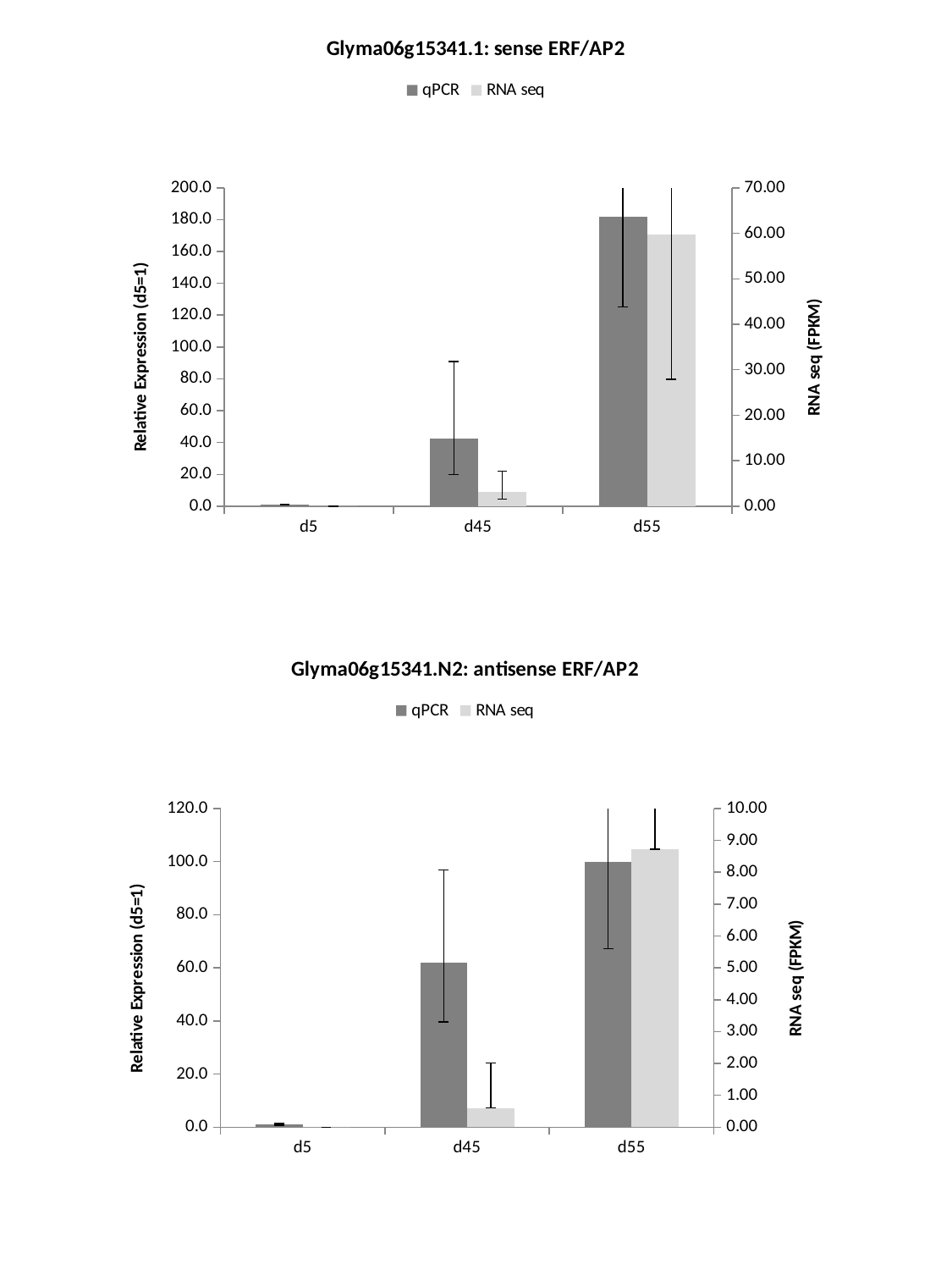

### Chart: Glyma06g15341.1: sense ERF/AP2
| Category | | | | |
|---|---|---|---|---|
| d5 | 1.0 | 0.0 | 0.0 | 0.0 |
| d45 | 42.52253243528438 | 0.0 | 0.0 | 3.08768 |
| d55 | 181.7416074599433 | 0.0 | 0.0 | 59.8098 |
### Chart: Glyma06g15341.N2: antisense ERF/AP2
| Category | | | | |
|---|---|---|---|---|
| d5 | 1.0 | 0.0 | 0.0 | 0.0 |
| d45 | 61.96230523435406 | 0.0 | 0.0 | 0.602578 |
| d55 | 100.0316368384266 | 0.0 | 0.0 | 8.72347 |

## Slide 3
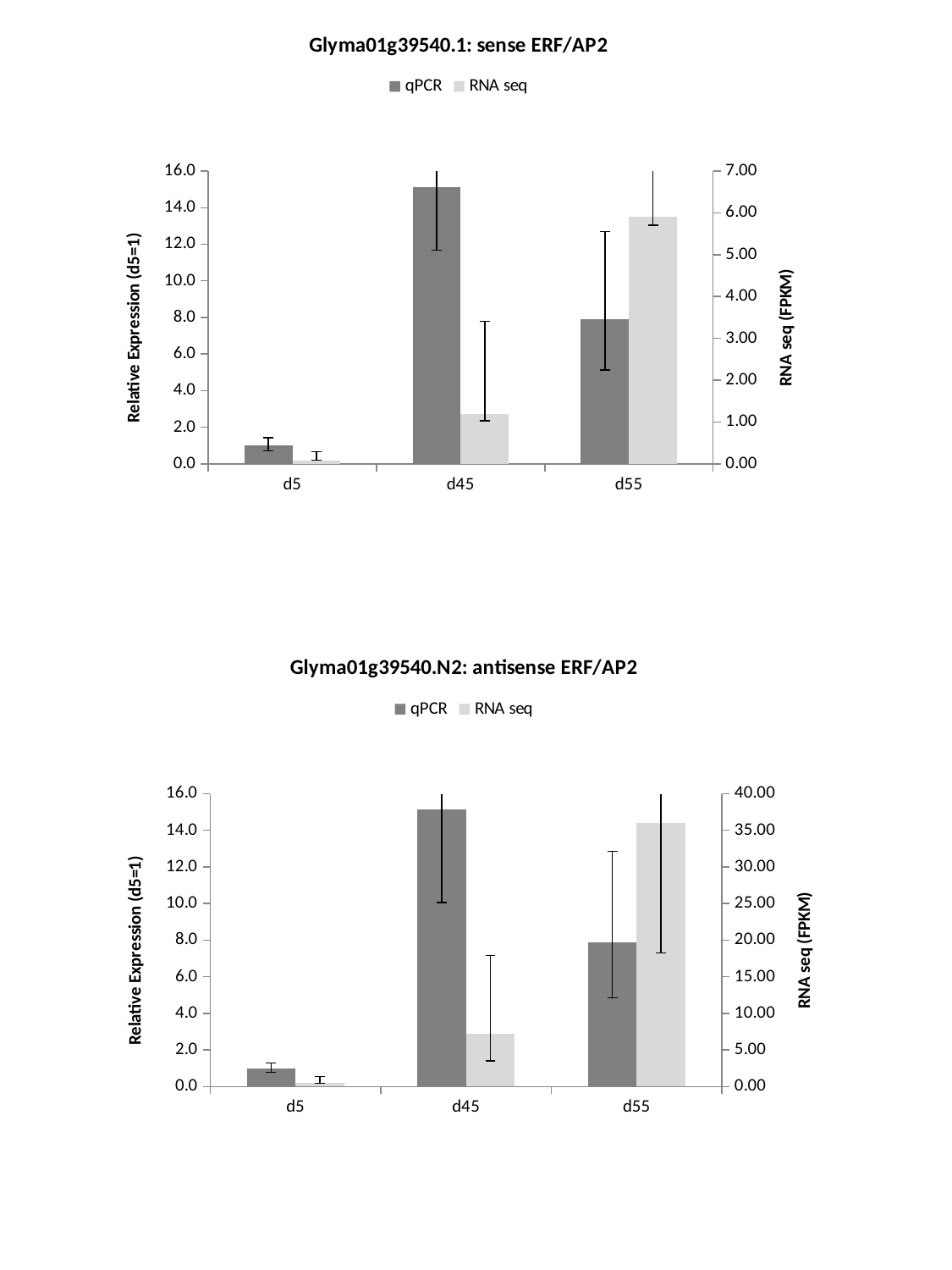

### Chart: Glyma01g39540.1: sense ERF/AP2
| Category | | | | |
|---|---|---|---|---|
| d5 | 1.0 | 0.0 | 0.0 | 0.083208 |
| d45 | 15.12864039638228 | 0.0 | 0.0 | 1.19087 |
| d55 | 7.887481229232385 | 0.0 | 0.0 | 5.91077 |
### Chart: Glyma01g39540.N2: antisense ERF/AP2
| Category | | | | |
|---|---|---|---|---|
| d5 | 1.0 | 0.0 | 0.0 | 0.461891 |
| d45 | 15.12864039638228 | 0.0 | 0.0 | 7.184129999999998 |
| d55 | 7.887481229232385 | 0.0 | 0.0 | 36.0513 |

## Slide 4
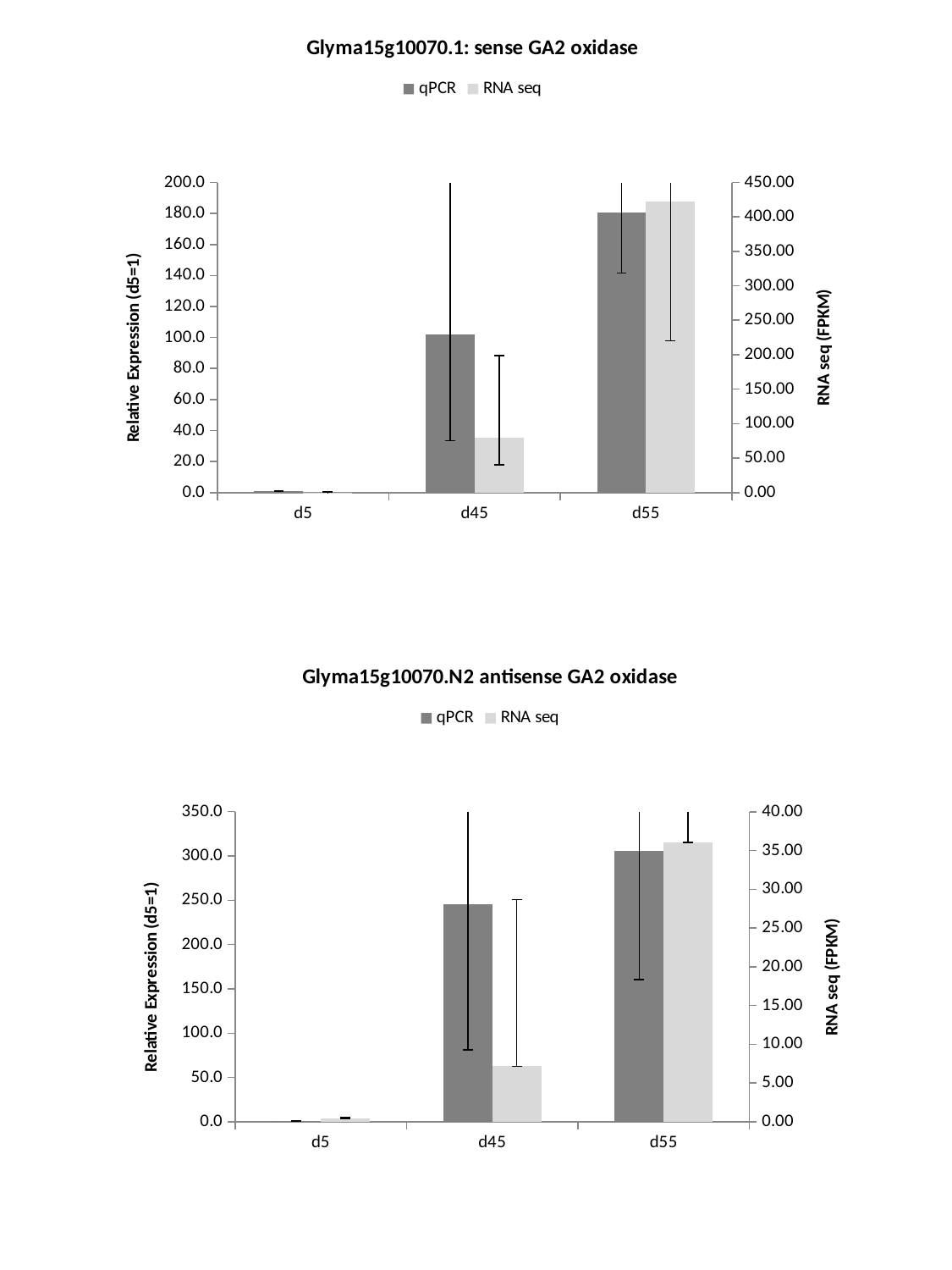

### Chart: Glyma15g10070.1: sense GA2 oxidase
| Category | | | | |
|---|---|---|---|---|
| d5 | 1.0 | 0.0 | 0.0 | 0.304242 |
| d45 | 101.883706910742 | 0.0 | 0.0 | 79.05829999999997 |
| d55 | 180.7547609037812 | 0.0 | 0.0 | 422.528 |
### Chart: Glyma15g10070.N2 antisense GA2 oxidase
| Category | | | | |
|---|---|---|---|---|
| d5 | 1.0 | 0.0 | 0.0 | 0.461891 |
| d45 | 245.6372962989733 | 0.0 | 0.0 | 7.184129999999998 |
| d55 | 306.058791208464 | 0.0 | 0.0 | 36.0513 |

## Slide 5
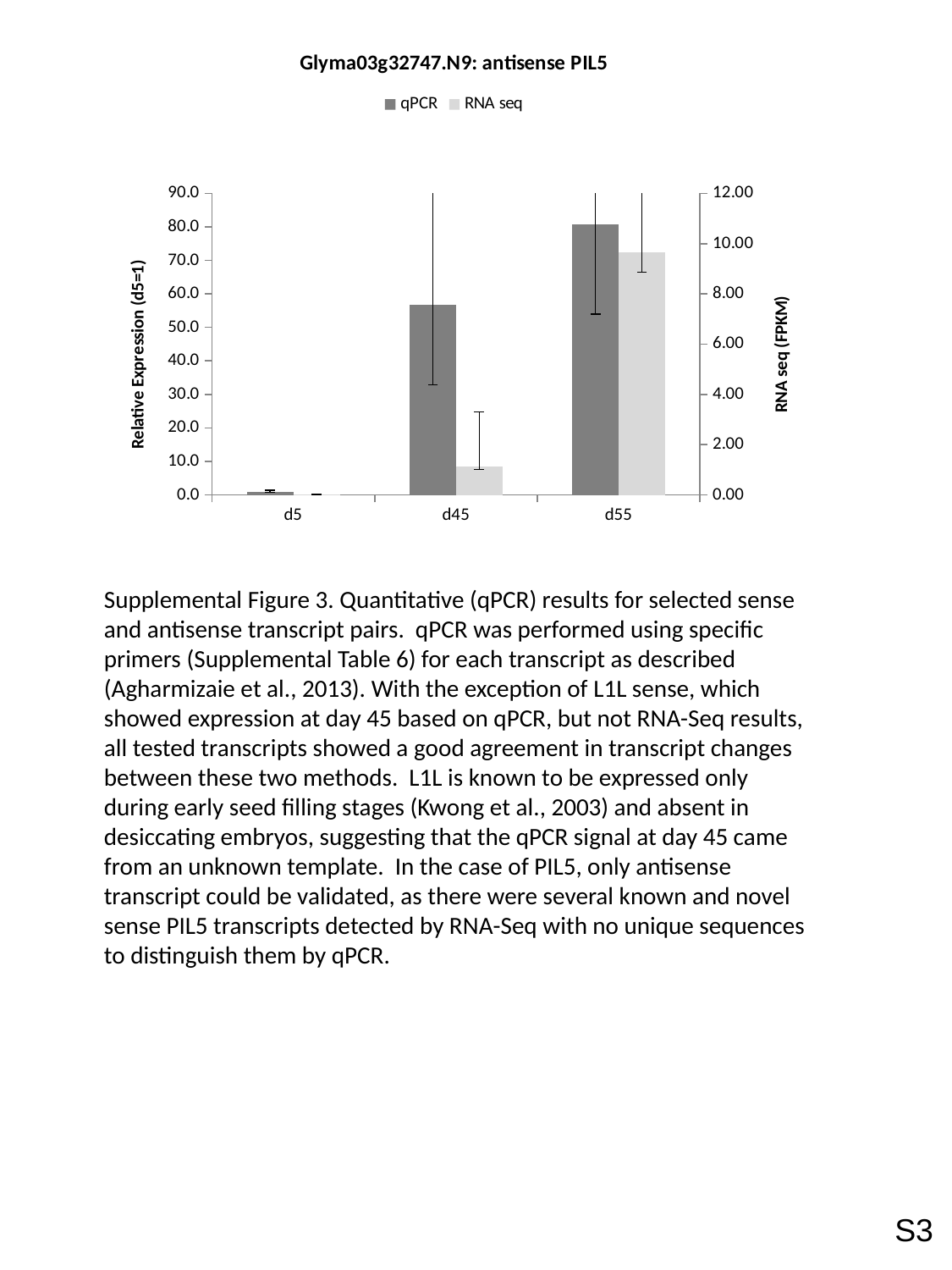

### Chart: Glyma03g32747.N9: antisense PIL5
| Category | | | | |
|---|---|---|---|---|
| d5 | 1.0 | 0.0 | 0.0 | 0.00520532 |
| d45 | 56.68827682400033 | 0.0 | 0.0 | 1.14195 |
| d55 | 80.82916685431658 | 0.0 | 0.0 | 9.648779999999999 |Supplemental Figure 3. Quantitative (qPCR) results for selected sense and antisense transcript pairs. qPCR was performed using specific primers (Supplemental Table 6) for each transcript as described (Agharmizaie et al., 2013). With the exception of L1L sense, which showed expression at day 45 based on qPCR, but not RNA-Seq results, all tested transcripts showed a good agreement in transcript changes between these two methods. L1L is known to be expressed only during early seed filling stages (Kwong et al., 2003) and absent in desiccating embryos, suggesting that the qPCR signal at day 45 came from an unknown template. In the case of PIL5, only antisense transcript could be validated, as there were several known and novel sense PIL5 transcripts detected by RNA-Seq with no unique sequences to distinguish them by qPCR.
S3
