# Supplementary material for: Transcriptome-wide functional characterization reveals novel relationships among differentially expressed transcripts in developing soybean embryos
Source: BMC Genomics. 2015 Nov 14;16:928. doi: 10.1186/s12864-015-2108-x (PMC4647491; doi:10.1186/s12864-015-2108-x)
Supplement: Additional file 9: Figure S4. — Distribution of 2,938 transcripts among the super-clusters. The graph shows the distribution of known and novel transcripts belonging to different Cuffcompare classes among six super-clusters. (PPTX 367 kb) [file 12864_2015_2108_MOESM9_ESM.pptx]

## Slide 1
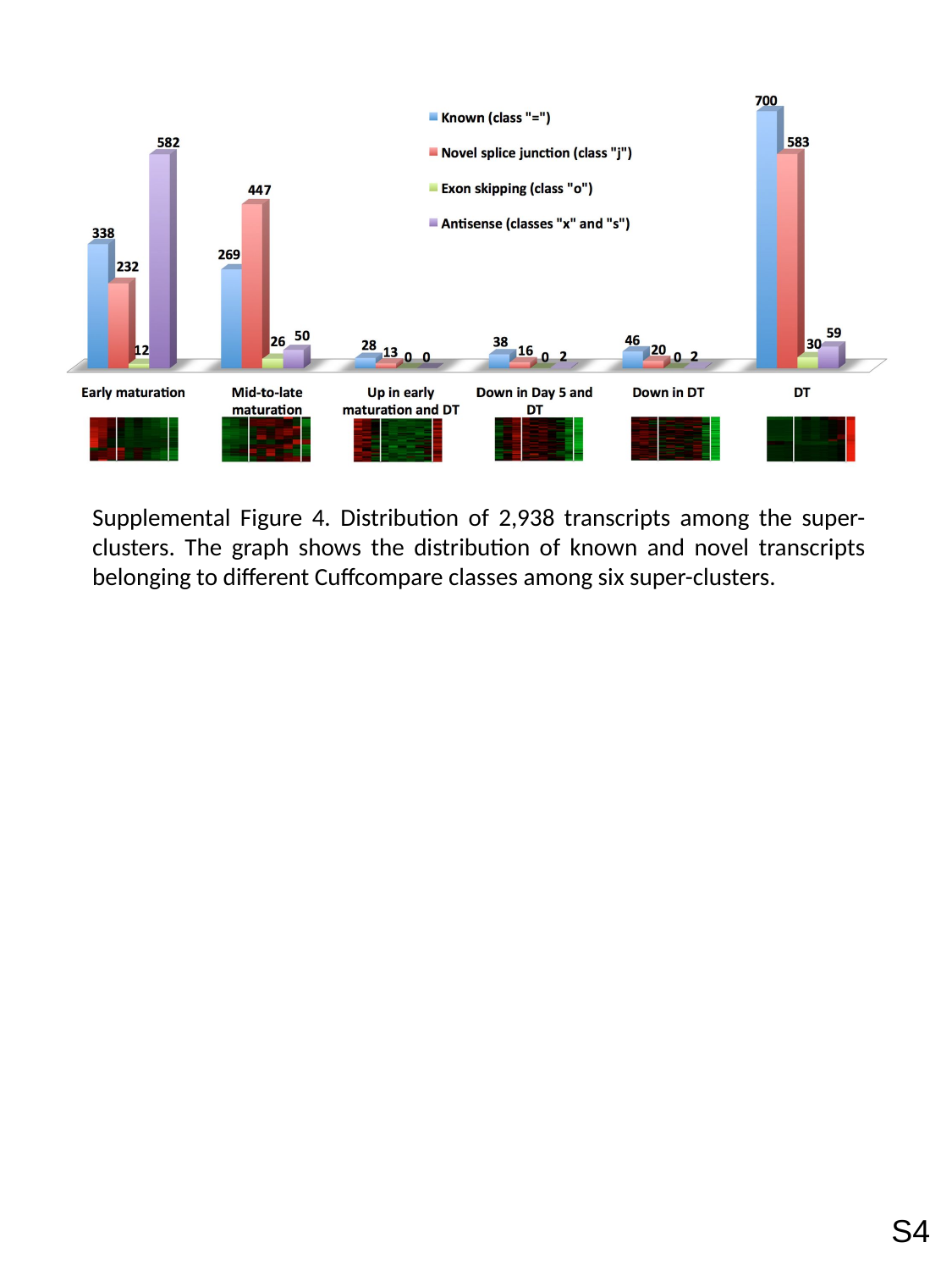

Supplemental Figure 4. Distribution of 2,938 transcripts among the super-clusters. The graph shows the distribution of known and novel transcripts belonging to different Cuffcompare classes among six super-clusters.
S4
