# Supplementary material for: Transcriptome-wide functional characterization reveals novel relationships among differentially expressed transcripts in developing soybean embryos
Source: BMC Genomics. 2015 Nov 14;16:928. doi: 10.1186/s12864-015-2108-x (PMC4647491; doi:10.1186/s12864-015-2108-x)
Supplement: Additional file 12: Figure S5. — The participation of transcripts in proposed signaling pathways involved in ABA- and FUS3-related responses. (A) ABA responsive SVs unique to DT with associated functions. NCED5 probably synthesizes ABA during DT. RCAR3 is a well-studied component of ABA receptor complex leading to signaling mediated by several ABA-related components subjected to AS, although their relationships and interactions are not clear (red arrows). GCR1 is an ABA responsive G protein-coupled receptor that was connected to 38 transcripts, some of which are already known to be associated with ABA signaling leading to dormancy (Fig. 9a and b). Possible interactions between the two receptors are not established as yet. (B) FUS3 regulon of transcripts related to the inferred B3 network in developing soybean embryos. These signaling pathways were drawn in the Beacon editor. Signaling components subjected to AS are shown in black boxes accompanying the relevant activities. (PPTX 303 kb) [file 12864_2015_2108_MOESM12_ESM.pptx]

## Slide 1
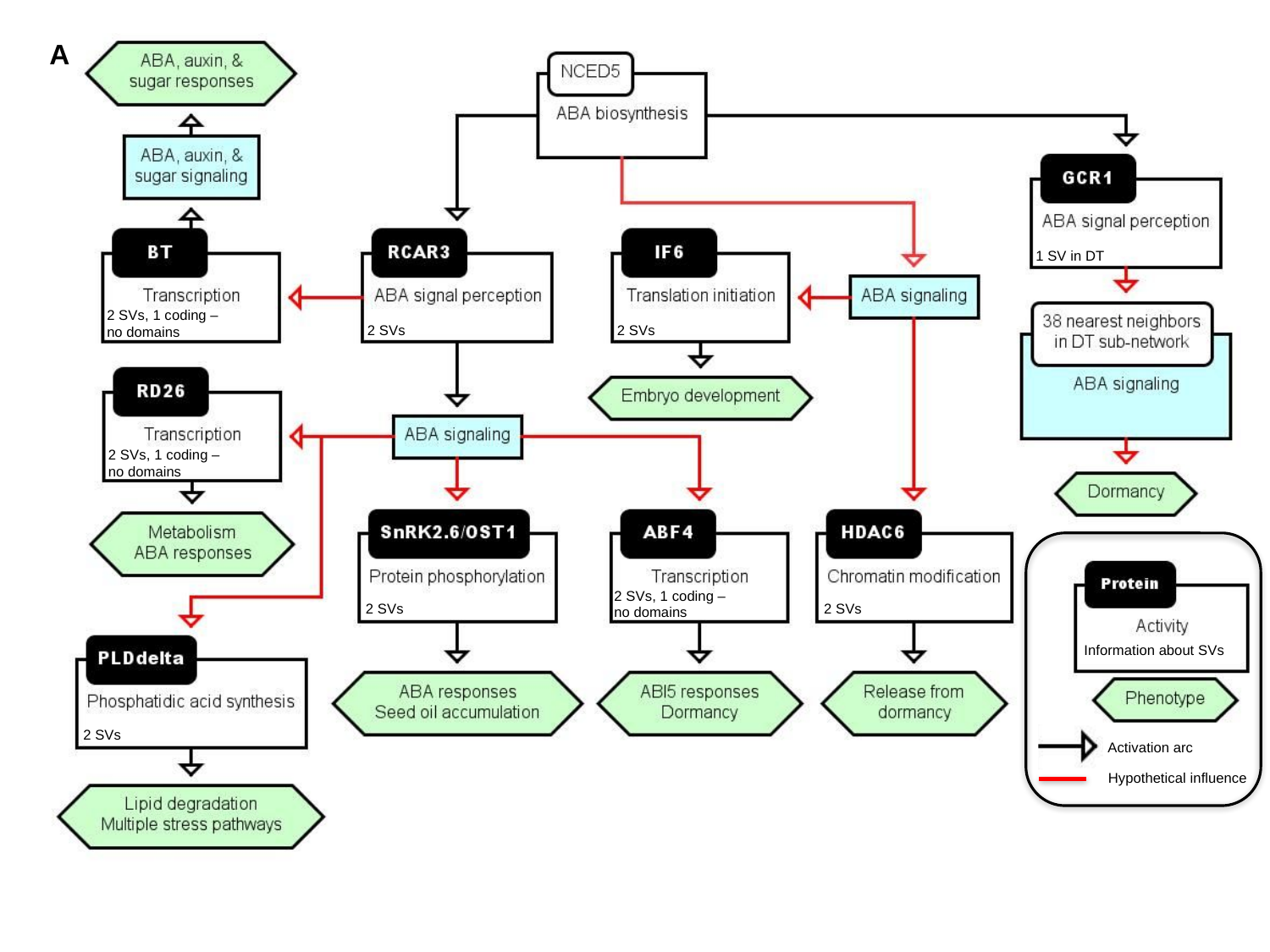

A
1 SV in DT
2 SVs, 1 coding – no domains
2 SVs
2 SVs
2 SVs, 1 coding – no domains
2 SVs, 1 coding – no domains
2 SVs
2 SVs
Information about SVs
2 SVs
Activation arc
Hypothetical influence

## Slide 2
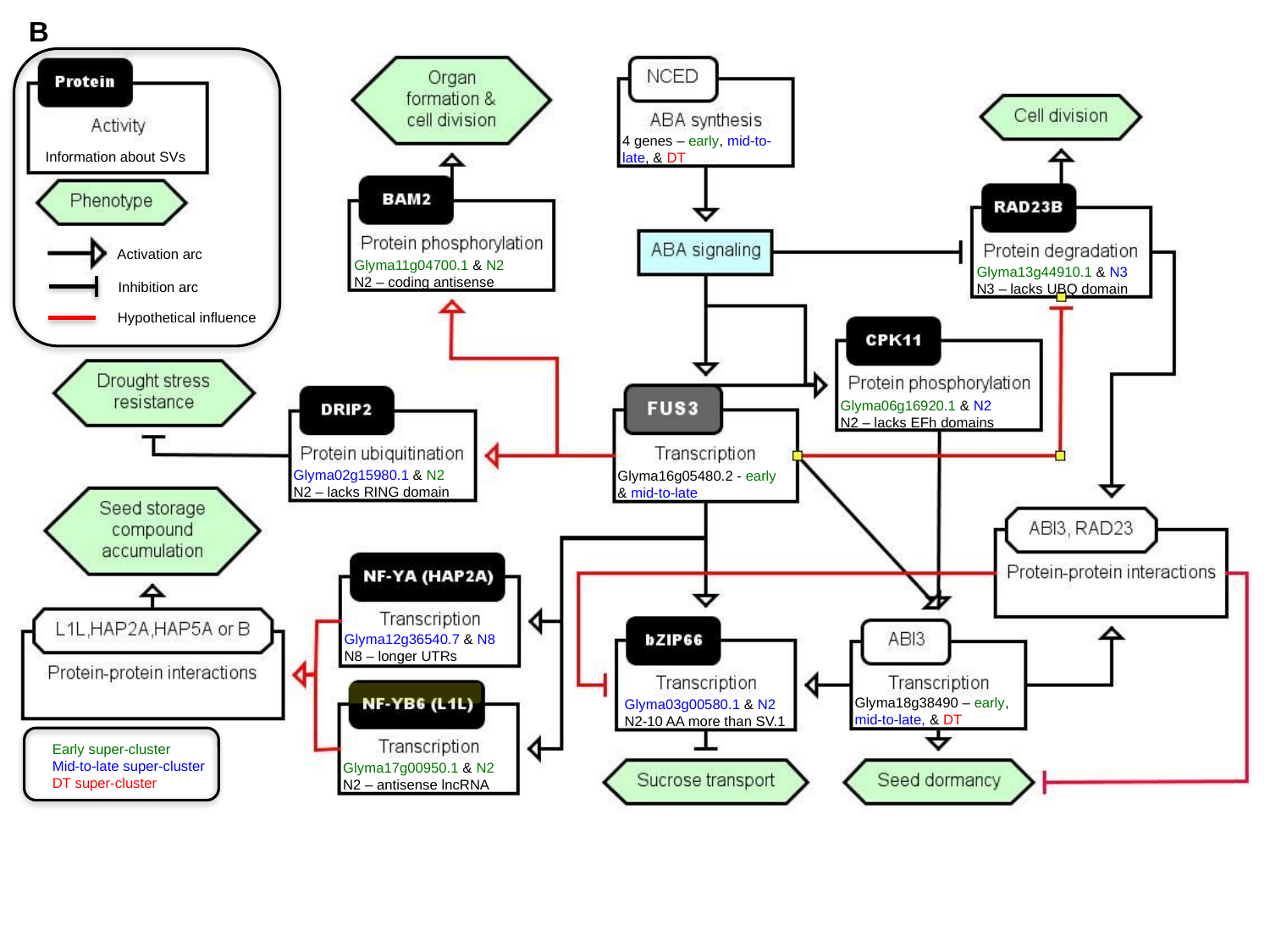

B
4 genes – early, mid-to-late, & DT
Information about SVs
Activation arc
Glyma11g04700.1 & N2
N2 – coding antisense
Glyma13g44910.1 & N3
N3 – lacks UBQ domain
Inhibition arc
Hypothetical influence
Glyma06g16920.1 & N2
N2 – lacks EFh domains
Glyma02g15980.1 & N2
N2 – lacks RING domain
Glyma16g05480.2 - early & mid-to-late
Glyma12g36540.7 & N8
N8 – longer UTRs
Glyma18g38490 – early, mid-to-late, & DT
Glyma03g00580.1 & N2
N2-10 AA more than SV.1
Early super-cluster
Mid-to-late super-cluster
DT super-cluster
Glyma17g00950.1 & N2
N2 – antisense lncRNA

## Slide 3
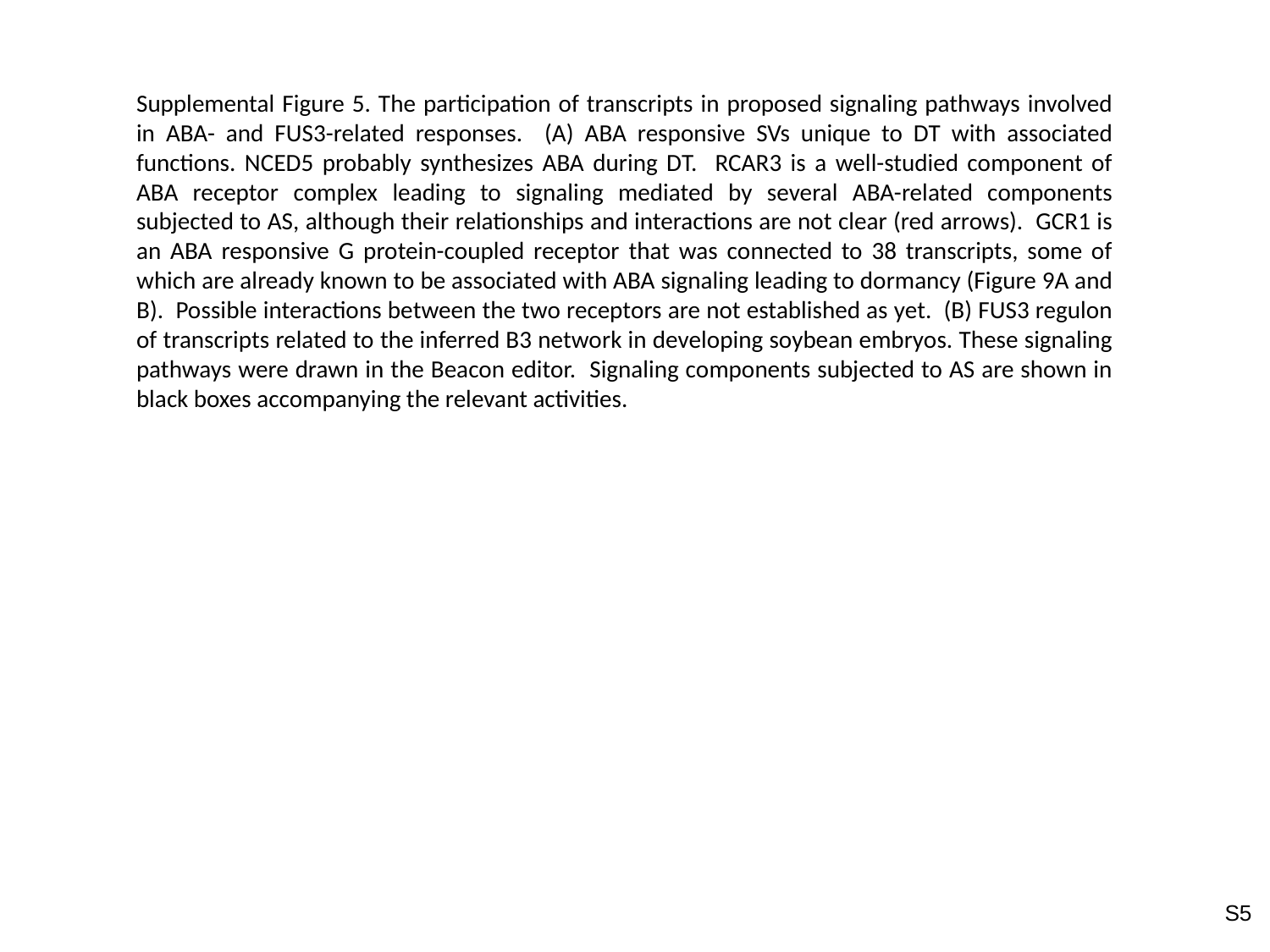

Supplemental Figure 5. The participation of transcripts in proposed signaling pathways involved in ABA- and FUS3-related responses. (A) ABA responsive SVs unique to DT with associated functions. NCED5 probably synthesizes ABA during DT. RCAR3 is a well-studied component of ABA receptor complex leading to signaling mediated by several ABA-related components subjected to AS, although their relationships and interactions are not clear (red arrows). GCR1 is an ABA responsive G protein-coupled receptor that was connected to 38 transcripts, some of which are already known to be associated with ABA signaling leading to dormancy (Figure 9A and B). Possible interactions between the two receptors are not established as yet. (B) FUS3 regulon of transcripts related to the inferred B3 network in developing soybean embryos. These signaling pathways were drawn in the Beacon editor. Signaling components subjected to AS are shown in black boxes accompanying the relevant activities.
S5
